# Dimeric Polyphenol Effect on Liquid-Ordered and Liquid-Disordered Membranes: Combined Insights from Molecular Dynamics Simulation and Langmuir Balance Measurements

**DOI:** 10.1021/acs.langmuir.5c05280

**Published:** 2026-03-20

**Authors:** Ruifeng Wang, Suvi Heinonen, Elina Vuorimaa-Laukkanen, Chunmei Li, Tapani Viitala, Alex Bunker

**Affiliations:** † College of Food Science and Technology, 47895 Huazhong Agricultural University, Wuhan 430070, China; ‡ Division of Pharmaceutical Biosciences, Faculty of Pharmacy, 3835 University of Helsinki, Helsinki 00014, Finland; § State Key Laboratory of Quality Research in Chinese Medicine, Institute of Chinese Medical Sciences, 59193 University of Macau, Macau 999078, China; ∥ Chemistry and Advanced Materials, Faculty of Engineering and Natural Sciences, Tampere University, Tampere 33720, Finland; ⊥ Division of Pharmaceutical Chemistry and Technology, Faculty of Pharmacy, University of Helsinki, Helsinki 00014, Finland; # Pharmaceutical Sciences Laboratory, Faculty of Science and Engineering, Åbo Akademi University, Turku 20520, Finland

## Abstract

Dimeric polyphenols have been postulated to be more effective in preventing obesity than monomeric ones; however, the mechanism through which polyphenols act on cells remains poorly understood. A leading hypothesis is the inactivation of the intracellular lipid synthesis signaling pathway that leads to lipid accumulation in 3T3-L1 preadipocytes through interference with the signaling activity of the raft domains of their plasma membranes. Thus, understanding the behavior of polyphenols on the membrane surface is essential to elucidating the mechanism through which they disrupt lipid raft structures differently. In this study, we first established two bilayer membrane models of different composition, the liquid-ordered (Lo) membrane containing cholesterol and sphingomyelin representing a raft domain, and the liquid-disordered (Ld) membrane containing phosphatidylcholine representing a nonraft domain; the surface behavior and consequent interaction of three representative polyphenols (one, a monomeric phenol and the other two, dimeric phenols) with the two bilayer membrane models were then investigated, computationally, through molecular dynamics simulations. The dimeric polyphenols were found to bind to both the Ld and Lo membranes through extensive hydrogen bonding, for the case of the Ld membrane penetrating deeper within the membrane than monophenol, but for the case of the Lo membrane locating predominantly to the lipid headgroups similar to monophenol. Moreover, the dimeric polyphenols exhibited significantly prolonged binding times in both Ld and Lo membranes with a more pronounced effect observed in the Lo membrane. Finally, Langmuir film balance measurements were performed to provide complementary evidence and support the results from the simulations.

## Graphical abstract

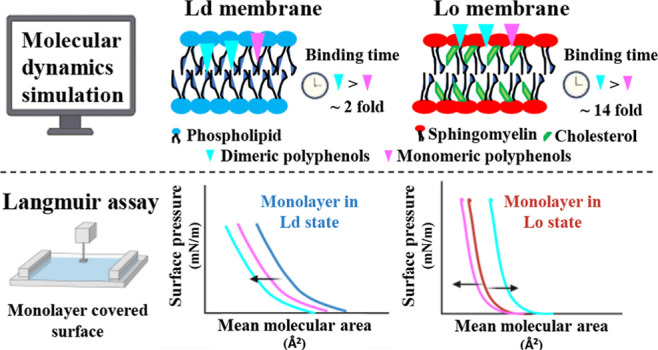

## 1 Introduction

Polyphenols have been postulated to be effective in preventing obesity by evidence obtained from cell culture, animal, and human models.^1,2^ A previous study by our group found that two dimeric polyphenols that possess a galloyl moiety, epicatechin-3-gallate-(4β → 8, 2β → → 7)-epicatechin-3-gallate (ECG dimer) and epigallocatechin-3-gallate-(4β → 8, 2β → → 7)-epigallocatechin-3-gallate (EGCG dimer), were shown to significantly inhibit lipid accumulation in 3T3-L1 preadipocytes by inactivating the intracellular lipid synthesis signaling pathway.^3^ As a result, they were considered to be the polyphenols possessing strong differentiation-inhibitory ability in 3T3-L1 preadipocytes, with the half-maximal inhibitory concentrations of 20.36 and 46.53 μM, respectively.^4^ While an extensive study of the mechanisms of inhibition of their intracellular signaling pathways has been carried out by several research groups,^5,6^ the understanding of how dimeric polyphenols act on cells remains poorly understood.

According to the raft hypothesis, cell membranes consist of liquid-ordered (Lo) and liquid-disordered (Ld) membrane fractions, with Lo domains corresponding to lipid rafts in biological membranes; this two-phase nature of the membrane plays an important role in 3T3-L1 preadipocyte differentiation.^7,8^ In particular, the Lo domains, enriched in cholesterol, sphingolipids, and signaling proteins, perform a signaling transduction function during induction of the differentiation of 3T3-L1 preadipocytes.^9,10^ For example, the structural integrity of the Lo domains was found to be necessary for transmitting the signal intracellularly and thereby initiating the insulin receptor-mediated lipid synthesis signaling pathway in 3T3-L1 preadipocytes.^10–12^ Hence, such disruption of the Lo domain structure has, in the past, been seen as an important mechanism to inhibit 3T3-L1 preadipocyte differentiation.^13^ Several studies have confirmed this hypothesis and found that some polyphenols with galloyl groups can significantly disrupt the structure of lipid rafts by affecting cell membrane fluidity^14^ and cholesterol binding.^5^ Importantly, polyphenols that are dimerized stand out significantly, exhibiting notable capabilities in inhibiting preadipocyte differentiation and disrupting the structure of lipid rafts. However, the mechanism through which this occurs is not completely understood. Given that the cell membrane is the initial point of contact for dimeric polyphenols with the cell, understanding their behavior on the membrane surface is essential to elucidating the role they play in disrupting lipid raft structures and thus exerting the observed strong bioactivity. Molecular dynamics (MD) simulation provides a means to obtain atomistic level insights that can elucidate this phenomenon.^15^ For example, this technique has been successfully applied in the past to explore the details of interactions between lipid raft components,^16^ the nature of lipid raft formation,^17^ and the interactions of active molecules with membrane rafts.^18^

Therefore, in this study, we established two different bilayer membrane models to represent the two different Lo and Ld domains. The MD simulations were combined synergistically with Langmuir monolayer experiments to investigate the surface behavior of the dimeric polyphenols in different membrane models. This work will contribute to a more comprehensive understanding of the mechanism through which dimeric polyphenols inhibit 3T3-L1 preadipocyte differentiation.

## 2 Methods

### 2.1 Construction for 3T3-L1 Preadipocyte Membrane Structural Domains

The lipid compositions of 3T3-L1 preadipocyte cell membranes have been measured and analyzed by Sánchez-Wandelmer et al. through a combination of high-performance liquid chromatography and thin-layer chromatography technology.^12^ The data showed that 3T3-L1 cell membranes were mainly composed of cholesterol, sphingomyelin, phosphatidylcholine, and phosphatidylethanolamine, accounting for 87% of the total membrane lipids. Further membrane fraction analysis indicated that sphingomyelin and cholesterol in a molar ratio of 1:2 is the composition of the liquid-ordered (Lo) fraction of the bilayer membrane, which represents lipid raft domains found in cell membranes, while phosphatidylcholine with unsaturated acyl chains was found to be the main constituent in the liquid-disordered (Ld) bilayer membrane, representing nonraft regions; the chemical structures of these lipids are shown in Figure 1. Thus, we chose 1-palmitoyl-2-oleoyl-*glycero*-3-phosphocholine (POPC) as the lipid for the Ld extracellular leaflet membrane model, with 40 POPC lipid molecules in each leaflet, which was chosen considering computational efficiency. For the Lo bilayer membrane model, we chose *N*-palmitoyl-d-erythrosphingosylphosphorylcholine (PSM) and cholesterol (CHOL), with 40 PSM and 80 CHOL in each leaflet, which was chosen with a molar ratio of 1:2 matching the experimental results. Thus, we chose these two different structural membrane models to represent the domains in the 3T3-L1 preadipocyte membrane. Subsequently, these different membrane models were constructed by the CHARMM-GUI Membrane Builder (http://www.charmm-gui.org/).^19^ Meanwhile, the CHARMM36 lipid force field was used for these membrane lipid models.^20–22^

**Figure 1:**
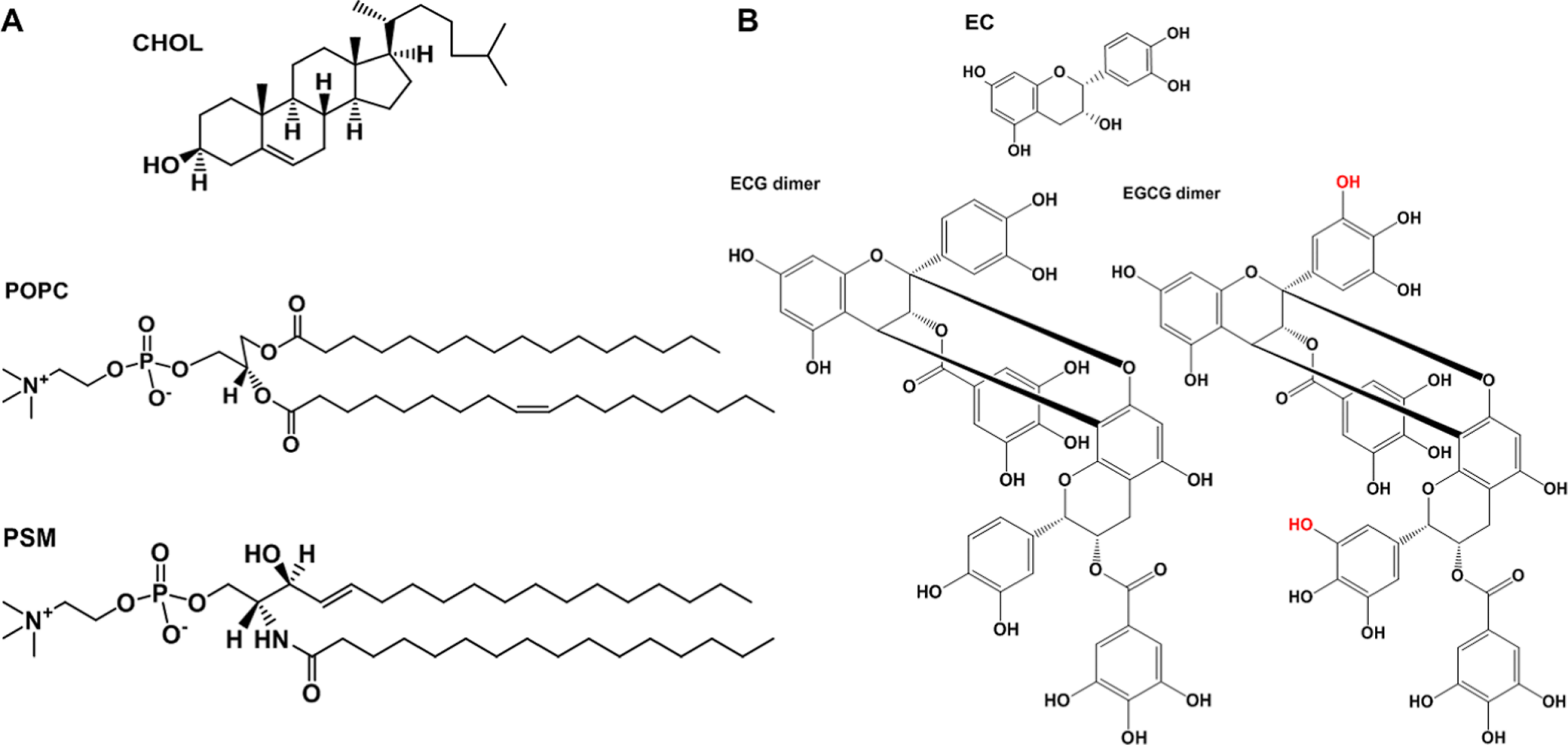
2D molecular structure of 3T3-L1 preadipocyte membrane lipids (A) and polyphenols (B) used in the present study: 1-palmitoyl-2-oleoyl-*sn*-glycero-3-phosphocholine (POPC), *N*-palmitoylsphingomyelin (PSM), cholesterol (CHOL), epicatechin (EC), epicatechin-3-gallate-(4β → 8, 2β → O → 7)-epicatechin-3-gallate (ECG dimer), and epigallocatechin-3-gallate-(4β → 8, 2β → O → 7)-epigallocatechin-3-gallate (EGCG dimer). The hydroxyl groups on the EGCG dimer are marked in red to distinguish its structural differences from the ECG dimer.

The molecular composition details of the two different membrane models can be found in Tables S1 and S2 (Supporting Information).

### 2.2 Molecular Dynamic Simulation Details

First, the two membrane models (Lo and Ld membranes) were solvated using TIP3P as the water model.^23,24^ Next, the models were equilibrated using the Parrinello–Rahman barostat for 100 ns under physiological conditions at constant pressure (1 bar) and temperature (310 K) using the GROMACS 2022 software package.^25^ Subsequently, the final 50 ns of the trajectory from the membrane equilibrium simulation was used to calculate and compare the area per lipid (APL) and membrane thickness of different membranes using FATSLiM software.^26^ The lateral diffusion coefficients (*D*_*xy*_) were obtained via linear fit to the phosphorus (POPC, PSM) or hydroxyl oxygen (cholesterol) atoms’ mean square displacements (MSD) by using the LiPyphilic^27^ together with MDAnalysis.^28,29^ The membrane equilibrium simulations were extended so that the final 500 ns of the simulation could be used for the analysis. Based on the MSD plots (Figure S1, Supporting Information), the fitting was performed between lagtimes τ = 10 ns and τ = 200 ns. To investigate the relationship between lipid mobility and polyphenol-lipid interactions (i.e., *D*_*xy*_ vs *N*_contacts_), we calculated the number of polyphenol-lipid contacts between heavy atoms within a 4.5 Å cutoff for each lipid or lipid subregion (choline, phosphate, glycerol, tails). The contact analysis was performed using the same extended trajectory data as the *D*_*xy*_ calculations, and the resulting contact counts were correlated with the average *D*_*xy*_ values from the same membrane leaflet (distributions are shown in Figure S2, Supporting Information).

Our previous study correlated the differentiation-inhibitory activity of polyphenols on 3T3-L1 preadipocytes and their interaction parameters with lipid rafts and then obtained different activity classes through cluster analysis.^4^ Thus, in the present study, we chose two dimeric polyphenols as representatives of high inhibitory-activity polyphenols: (1) epicatechin-3-gallate-(4β → 8, 2β → O → 7)-epicatechin-3-gallate (ECG dimer) and 2) epigallocatechin-3-gallate-(4β → 8, 2β → → 7)-epigallocatechin-3-gallate (EGCG dimer). Meanwhile, epicatechin (EC) was used as a control to represent the low inhibitory-activity polyphenol. To construct the topology files of the polyphenols for our MD simulation study, first, their 2D chemical structure (EC, ECG dimer, and EGCG dimer) was generated using the ChemBioDraw module of the ChemBioffice software 12.0 (Cambridge soft, MA, USA) (chemical structures shown in Figure 1). From these 2D structures, a rough geometric optimization was performed by using the Chem3D module. Subsequently, the 3D chemical structure was obtained using *MM2* job, that is the 3D conformation optimized program of Chem3D. In order to more accurately reveal the structural details of these polyphenols, geometry optimization was performed utilizing the Hartree–Fock (HF) theory with the 6–31G­(d,p) basis set using the GAUSSIAN 09 software package.^30^ After geometry optimization, the restrained electrostatic potential (RESP) charges were also fitted using the Multiwfn 3.8 software package.^31^ Subsequently, these polyphenols were uploaded to the CHARMM General Force Field server (https://cgenff.umaryland.edu/).^32^ The parameters of polyphenols were written into a toppar stream file. Finally, the topology files of these polyphenols were generated using the cgenff_charmm2gmx Python script, and the assigned charges were replaced by RESP charges.

For the complex polyphenols-membrane systems, polyphenols were separately inserted into the center of the water phase in each membrane model, and all systems were then solvated once again.

The numbers of water molecules added to different systems are found in Tables S1 and S2 (Supporting Information). Subsequently, these six systems were energy-minimized using the steepest descent algorithm, followed by equilibration under a canonical (*NVT*) ensemble for 1 ns at 310 K and equilibration under an isothermal–isobaric (*NPT*) ensemble for 5 ns at 1 bar and 310 K. For all the systems, production runs of over 500 ns at the same temperature and pressure were carried out and all simulations were replicated at least three times. The V-rescale thermostat with a coupling time constant of 0.1 ps was used for temperature coupling;^33^ the temperatures of the polyphenols, lipids, and water molecules were controlled independently. Pressure was controlled using the Parrinello–Rahman barostat with a semi-isotropic coupling scheme and a coupling time constant of 2 ps.^34^ The periodic boundary conditions were used for all directions, and the linear constraint solver algorithm was applied in constraining bonds connecting to hydrogen atoms.^35^ The cutoff for the Lennard-Jones interactions was set to 1.2 nm. The particle mesh Ewald method was used for electrostatic interactions,^36^ and the Coulomb cutoff was set to 1.2 nm. The equations of motion were integrated every 2 fs with the leapfrog algorithm.^37^ Finally, the simulation trajectories were visualized by VMD 1.9.2 software,^38^ and the interaction analyses were performed on the final 100 ns of the simulation. Standard deviations were obtained from the time averages of the three replicates. The lipid–lipid hydrogen bonds and hydrogen bonding between the polyphenols and lipids were quantified using the MDAnalysis package^28,39^ with the donor–acceptor distance cutoff of 3.0 Å and a donor-hydrogen-acceptor angle cutoff of 150°.

### 2.3 Langmuir Monolayer and Brewster Angle Microscopy

The ECG and EGCG dimers (purity: 95%) were isolated from persimmon based on a previously reported method.^40^ EC (purity: 98%) was purchased from Sigma-Aldrich Chemical Co. (St. Louis, MO) and CHOL, POPC, and PSM were purchased from Avanti Polar Lipids, Inc. (Alabaster, AL). To compare and validate the results from the simulations, Langmuir monolayers containing the respective lipids for the Ld and Lo monolayers were prepared. The surface pressure measurement was then performed using a KSV Mini through Langmuir film balance (KSV Instruments, Helsinki, Finland). A double barrier mini-Teflon trough with a total area of 24300 mm^2^ was used in compression isotherm experiments. Before each measurement, the trough and the barriers were cleaned using paper soaked in 2% Hellmanex and gently brushed with ethanol and then with water. Ultrapure water (Milli-Q IQ 7005 Ultrapure, Merck, Darmstadt, Germany) was used as the subphase and poured into the trough to a height of 2 mm above the trough edges and cleaned by aspiration of the water surface while the barriers were closed. After the barriers were opened after cleaning, the monolayers containing 1 mol/L of the respective lipids of the Ld (POPS) and Lo (CHOL and PSM with a molar ratio of 2:1) membranes were separately deposited onto the air–water interface using a Hamilton analytical syringe. The volume used was 25 μL. All lipids were dissolved in pure chloroform. After waiting 10 min for chloroform evaporation, the monolayers were then compressed at a compression rate of 10 mḿmin^–1^ while simultaneously measuring the surface pressure (π) as a function of the mean molecular area (A). The morphology of the films was also monitored with a Brewster angle microscope (BAM, KSV Optrel BAM300, KSV Instruments Ltd., Helsinki, Finland). The BAM instrument was equipped with a 10 mW HeNe laser (633 nm) that was linearly polarized in the plane of incidence by a Glan-Thompson polarizer. The reflection from the interface passes through a second Glan-Thompson polarizer and was collected by a CCD camera. The microscope was adjusted so that the background reflection from the bare air–water interface was minimal. The spatial resolution of the system was approximately 2 μm. To measure the effect of polyphenols on the surface pressure and the morphology of the monolayers, three polyphenols (EC, ECG dimer, and EGCG dimer) were completely dissolved in a chloroform/methanol 2:1 v/v mixture. Then various concentrations of polyphenols (0, 0.25, 0.50, 0.75 mol/lipid) in different monolayers were prepared; other processes were as described above. The experiments for each monolayer surface pressure–area (π–A) isotherms were performed at least three times, and all measurements were performed at room temperature. In this study, data were presented as mean ± standard deviation of three independent replications, and significant differences (*p* < 0.05) were explored using a one-way ANOVA test.

## 3 Results and Discussion

### 3.1 Comparison of Structural and Dynamic Properties of Ld and Lo Membranes

To verify that our model membranes are appropriate models of Ld nonraft and Lo raft domains, structural and dynamics properties of the two membrane model systems were calculated without the presence of polyphenols; these properties include APL, membrane thickness, and lateral diffusion coefficients, and the results are shown in Table 1. For the Ld membrane, the APL value and bilayer thickness for POPC were in line with previous results for pure POPC lipid bilayers,^41,42^ and the membrane was thus in the liquid-disordered phase. In contrast, the corresponding value of the APL for the Lo membrane was significantly lower due to the formation of the highly ordered structure that the combination of the CHOL and PSM form, this is the defining characteristic of lipid rafts that results from the network of hydrogen bonds that form between CHOL and PSM.^43,44^ This was also supported by our result that the Lo membrane also had a significantly higher bilayer thickness in comparison to that of the Ld membrane, composed solely of POPC. Furthermore, these strong interactions between CHOL for PSM also increase the bilayer rigidity, as revealed by our result for the area compressibility modulus; the compressibility modulus for the Ld membrane was 1088.39 × 10^–3^ N/m, while that for the Lo membrane was 3486.43 × 10^–3^ N/m, more than three times greater, in line with results obtained in a previous study.^17^ In addition, our results showed that the lateral diffusion coefficient of the Ld membrane was 1.20 × 10^–7^ cm^2^/s, in agreement with the experimentally measured value for the Ld domain reported in the literature (≈1 × 10^–7^ cm^2^/s),^17^ while our results for the lateral diffusion coefficients of PSM and CHOL in the Lo membrane were also in agreement with experimental results (≈1 × 10^–8^ cm^2^/s),^45^ *a* factor of 10 smaller than that of the Ld membrane. Lipid diffusion in the Lo lipid raft domain is known to be strongly suppressed, and this is postulated to play an important role in their function.^9^ In short, our results for the structural and dynamic properties of our membrane models indicate that they are suitable as representative of the 3T3-L1 preadipocyte membrane structural domains that we are modeling.

**Table 1:** Structural Properties of the Disordered Membrane (Ld) and Ordered Membrane (Lo) and the Effect of Polyphenols on Their Properties^a^ ^,^ ^b^ ^,^ ^c^

system 1	Ld membrane-polyphenols			
Composition	Ld domain	EC	ECG dimer	EGCG dimer
area per lipid (nm^2^)	0.651 ± 0.002	0.640 ± 0.003#	0.645 ± 0.003	0.640 ± 0.004#
bilayer thickness (nm)	3.90 ± 0.01	3.91 ± 0.02	3.90 ± 0.01	3.92 ± 0.03
lateral diffusion coefficients (10^–7^ cm^2^/s)	1.20 ± 0.24	0.72 ± 0.28#	0.58 ± 0.09*	0.78 ± 0.13#

system 2		Lo membrane-polyphenols			
Composition		Lo domain	EC	ECG dimer	EGCG dimer
area per lipid (nm^2^)		0.391 ±	0.387 ±	0.387 ±	0.387 ±
		0.001	< 0.001*	< 0.001*	< 0.001*
bilayer thickness (nm)		4.50 ± 0.01	4.51 ± 0.01	4.51 ± 0.01	4.52 ± 0.02
lateral diffusion coefficients (10^–7^ cm^2^/s)	PSM	0.07 ± 0.04	0.04 ± 0.01	0.07 ± 0.02	0.09 ± 0.08
	CHOL	0.08 ± 0.04	0.04 ± 0.01	0.07 ± 0.03	0.09 ± 0.08

a Note: results were expressed as mean ± standard deviation (*n* = 3 independent replicates).

b # represents *p* < 0.05 (vs pure membrane); * represents *p* < 0.01 (vs pure membrane).

c All analyses consider both membrane leaflets. (calculated results from the average of three values from single simulated frames).

### 3.2 Structural Effect of Polyphenols on Different Membranes

Having verified our membrane models, we calculated how their structural and dynamic properties are affected by the presence of three polyphenols that we were investigating; the results are also shown in Table 1. For our Ld model membrane, the APL was significantly decreased (*p* < 0.05), but bilayer thickness did not show significant changes when the polyphenols were added to the Ld membrane. The APL in the Lo model membrane also significantly decreased (*p* < 0.01) for the three polyphenols tested and bilayer thickness slightly increased by the presence of polyphenols, with no significant difference for the three polyphenols tested. This indicates that the extent to which polyphenols affect the Lo membrane seems to be the same. While the polyphenols affected APL and membrane thickness in a similar manner both in the Ld and Lo membranes, their effects on the dynamic properties of the membranes, as determined by measuring the lateral diffusion coefficient (*D*_*xy*_), were markedly different. For the Ld membrane, all polyphenols displayed a similar trend of reducing the *D*_*xy*_ on the same leaflet after treatment (Table 1, Figure S2a, Supporting Information). This trend mirrors the findings of a previous experimental study: the membrane fluidity was strongly constrained in the presence of active polyphenols.^14^ The experimental results indicated that the reduction in membrane fluidity was highly correlated to the inhibitory effect of 3T3-L1 preadipocyte differentiation. Thus, the strong 3T3-L1 preadipocyte differentiation inhibitory activity of the ECG dimer and EGCG dimer might be the result of a rapid reduction in the lateral mobility of the Ld fraction of the membrane. In addition, Mei et al. found a correlation between the strength of the binding of molecules to the membrane and the resulting decrease in the lateral diffusion coefficients of lipids in the membrane.^46^ Similarly, our data show a trend for a negative linear correlation between lipid mobility in the Ld membrane and the number of contacts formed by the polyphenols in the membrane headgroup region (Figure S3A, Supporting Information). Therefore, the results indicated that all tested polyphenols can bind to the Ld membrane, with the dimeric ECG polyphenol binding the strongest (lowest *D*_*xy*_ value), followed by the EGCG dimer. For the Lo membrane, EC, as a representative polyphenol with low-inhibitory activity, was found to shift the individual lipid *D*_*xy*_ values for PSM and CHOL homogeneously toward lower values (Table 1, Figure S2b, Supporting Information). Conversely, both ECG and EGCG dimers caused varying effects on the lateral mobility of lipids on the same leaflet and could both increase and decrease the *D*_*xy*_ depending on the replica (Figure S2b, Supporting Information). This effect was the most pronounced for the EGCG dimer, as exemplified by the distinctive distributions for each replica. We suspect that these diverging distributions arise from two primary sources of variability: (i) the different ways in which the dimeric polyphenols interact with the membrane polar region, enabled by their numerous hydroxyl groups and the various orientations they can adopt, and (ii) differences in the composition of the immediate lipid environment. This interpretation is supported by the substantial variability in the hydrogen-bonding patterns (Figure 2) and by the strong positive correlation (Pearson’s r = 0.80, *p* < 0.01) between the extent of polyphenol-cholesterol contacts and lipid mobility (Figure S3B, Supporting Information), both of which reflect the combined influence of polyphenol orientation and local lipid environment. Interestingly, our data suggest that introducing dimeric polyphenols to the membrane can both reduce lipid mobility when the extent of polyphenol-cholesterol contacts is low and increase *D*_*xy*_ with a higher number of contacts (Figure S3Bb, Supporting Information), mirroring the overall complexity of polyphenol-induced changes in lipid mobility in the Lo membrane. The behavior clearly deviates from our observations in the Ld membrane and from previous reports with simplified liposome models in the fluid state.^14^

**Figure 2:**
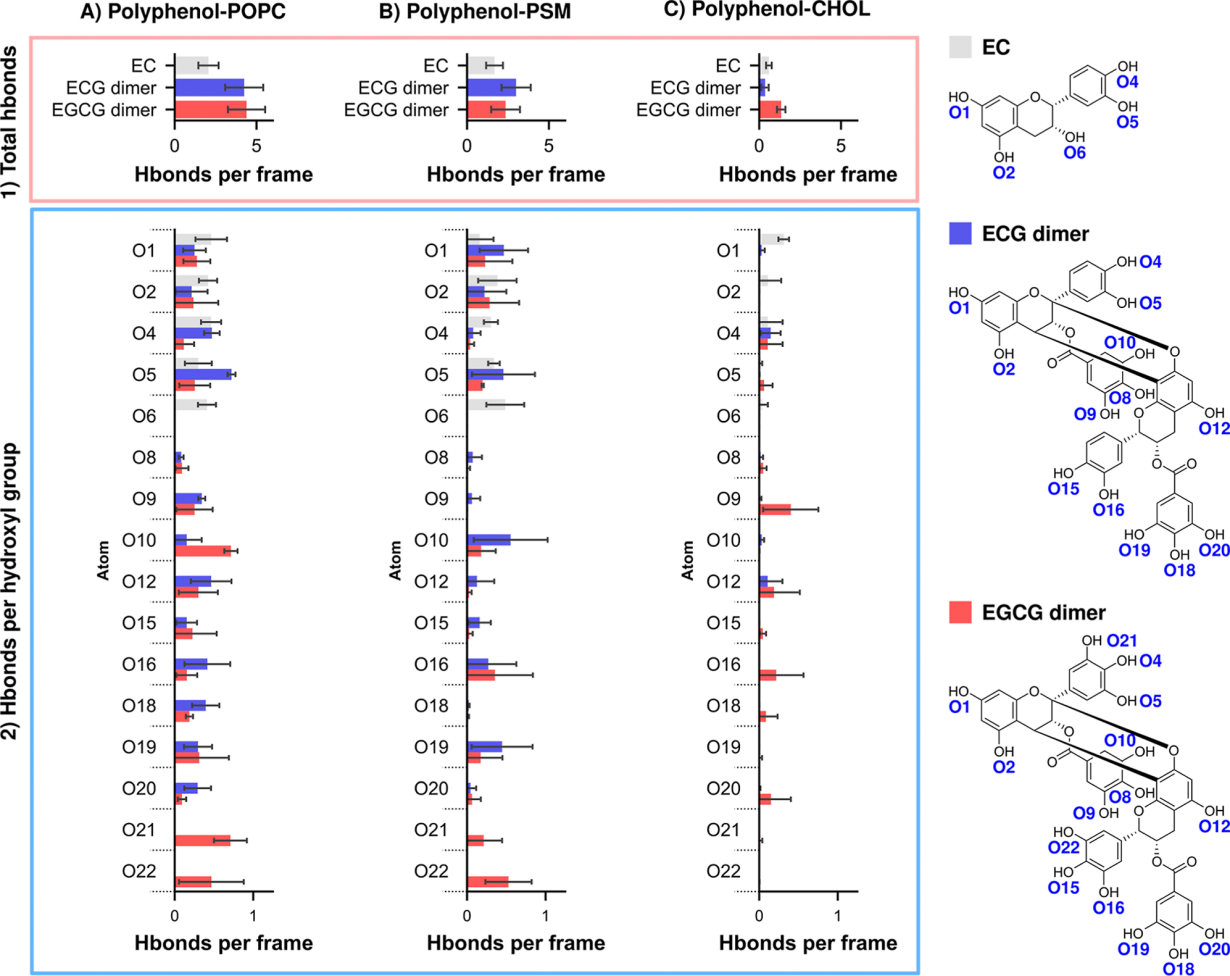
Hydrogen bonding between the polyphenols and the Ld or Lo membranes. The time averages of (1) total number of hydrogen bonds formed between polyphenols and lipid molecules and (2) the number of polyphenol-lipid hydrogen bonds for each hydroxyl group involved in the bond with standard deviations. In both cases, the number of hydrogen bonds has been assessed between polyphenols and (A) POPC, (B) PSM, or (C) CHOL. The hydroxyl groups have been named according to the index of their corresponding oxygen atom; the naming has been mapped to the polyphenols on the right side of the figure. Results were expressed as mean ± standard deviation (*n* = 3 independent replicates).

Since the hydrogen bonding network is known to play an important role in the formation of the Lo domain^43^ we investigated this next. In the Lo membrane, PSM can form multiple intermolecular hydrogen bonds with PSM or CHOL. This was supported by the time-averaged numbers of hydrogen bonds obtained from the trajectory (Table S3, Supporting Information). However, such interactions did not exist in the Ld membrane since the phosphate, carbonyl, and ether groups of POPC are only acceptors. Considering that polyphenols possess many hydroxyl groups, which can act both as hydrogen bond donors and as acceptors, the premise is that, in addition to forming intermolecular hydrogen bonds with the Ld and Lo membranes, the polyphenols also disrupt the hydrogen bond network formed within the Lo membrane. We measured the number of hydrogen bonds between polyphenols and the Ld or Lo membranes (Figure 2, Table S3, Supporting Information), and our results are in agreement with this hypothesis: in the Ld membrane, the average number of hydrogen bonds formed with the investigated polyphenols is 2.06 ± 0.62 for EC, 4.25 ± 1.16 for the ECG dimer, and 4.39 ± 1.14 for the EGCG dimer; the standard deviation of the results was too great to differentiate between the ECG and EGCG dimers. This signifies a substantially stronger binding of dimeric polyphenols with POPC than EC. For the case of the Lo membrane, there are the hydrogen bonds between the polyphenols and both the PSM and CHOL to consider, in addition to the effect on the intramembrane hydrogen bond network: the PSM–PSM and PSM-CHOL hydrogen bonds. Like the case for POPC in the Ld membrane, the dimerized polyphenols had a greater number of hydrogen bonds with PSM than with EC; the difference in this regard is particularly evident between EC (1.68 ± 0.52) and the ECG dimer (3.00 ± 0.90). In contrast, the result for CHOL was markedly different: EC and the ECG dimer formed a similar number of hydrogen bonds (0.59 ± 0.17 and 0.36 ± 0.22), whereas the EGCG dimer exhibited a significantly higher average number of hydrogen bonds (1.34 ± 0.25). Surprisingly, the additional hydroxyl groups found in the EGCG dimer (O21, O22, see Figure 2 for atom indexing) formed a negligible number of hydrogen bonds with cholesterol, and did not explain the difference. Moreover, no consistent pattern was observed for the hydroxyl groups involved in the hydrogen bond formation in the Lo membrane due to the high variability existing between replicas. This observation further supports the notion that the distinctive distributions of the individual lipid *D*_*xy*_ values in the Lo membrane possibly originate from the various molecular geometries adopted by the dimeric polyphenols upon membrane binding. Regarding the effect on the hydrogen bond network within the Lo membrane, the effect of the three polyphenols was the same: they all slightly lowered the number of intermolecular PSM–PSM hydrogen bonds and slightly raised the number of PSM-CHOL hydrogen bonds; polyphenols are thus seen to have the capacity to in some way affect the hydrogen bond network of the Lo membrane. Thus, even though the polyphenols do not seem to enter the membrane core of the Lo membrane to a sufficient extent to affect the APL, as is the case for the Ld membrane, our results show that they still affect the internal structure of the membrane.

### 3.3 Binding Behavior and Kinetics of Polyphenols to Different Membranes

The above results highlighted the strong binding of dimeric polyphenols to the membranes, followed by a pronounced effect on the structure of the membrane itself. Revealing the details on how polyphenols bind to the membranes will further contribute to explaining the disruptive effects of polyphenols on the structural and dynamic properties of membranes. The solvent accessible surface area (SASA) is a measure of the area of a particular molecule exposed to the solvent. The same measuring principle can be applied to determining the relative area of the polyphenol exposed to different areas of the membrane (i.e., the lipid headgroups and tails). This can be achieved through using the VMD SASA calculation tool,^47^ which effectively reveals the binding of polyphenols to a different region of the lipid membrane. Results of the portion of the surface area of the EC, ECG dimer, and EGCG dimer in contact with the lipid headgroups and tails are shown in Figure 3A,B. The SASA results show that for the case of all three polyphenols, they are predominantly in contact with the lipid headgroups for the case of the Lo membrane and predominantly in contact with the lipid tails for the case of the Ld membrane. This is in line with the APL results: in all cases, the polyphenols penetrate deeper into the looser structure of the Ld membrane than the tightly packed Lo membrane. This difference in polyphenol binding to membranes was also visualized by binding snapshots, as shown in Figure 3, where we can see the molecules sitting deeper within the Ld membrane and sitting at the surface for the case of the Lo membrane.

**Figure 3:**
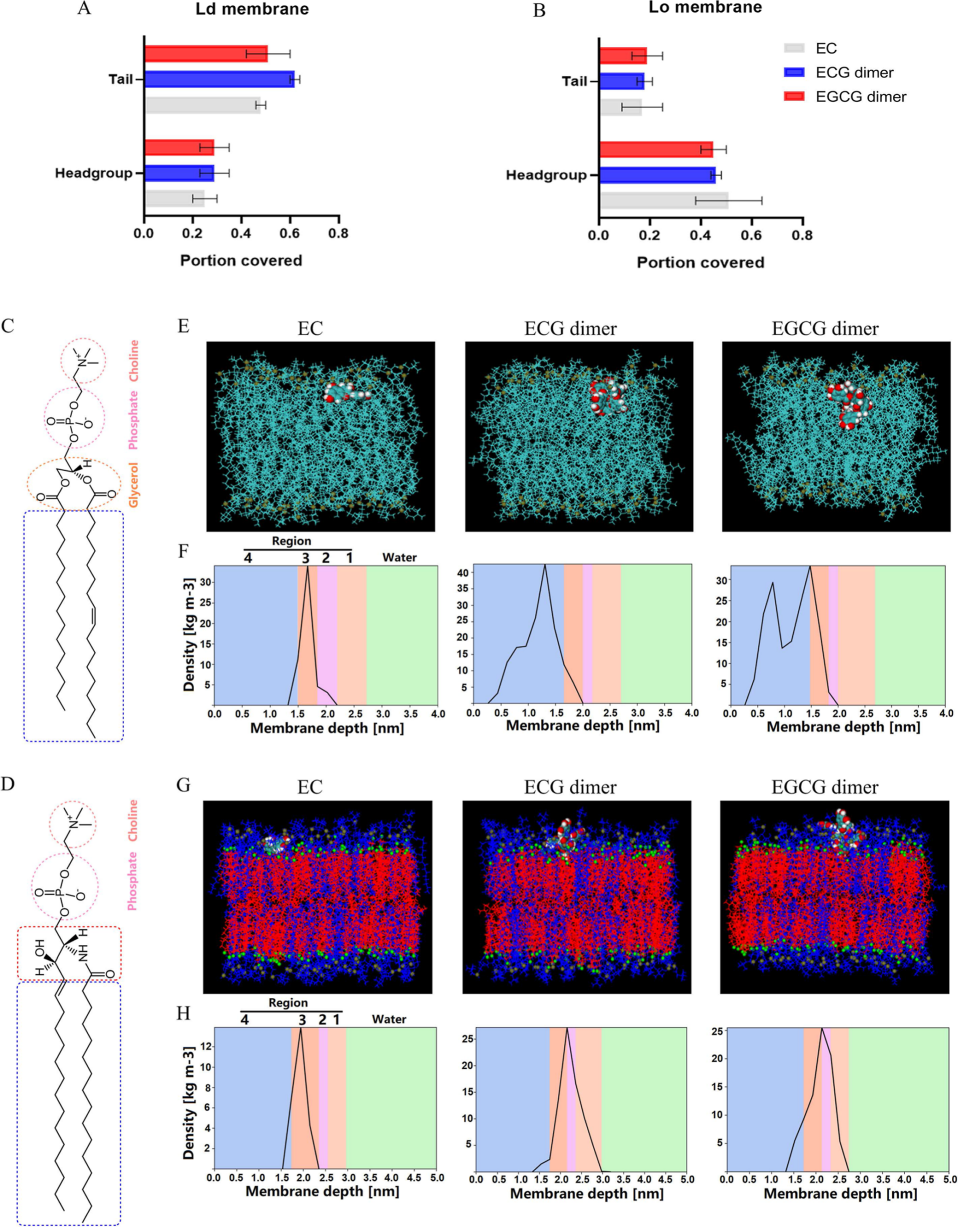
Overview of interactions between polyphenols and Ld as well as Lo membranes. Surface area of polyphenols covering the headgroup and tail of lipids in the Ld (A) and the Lo (B) membranes. Secondary building units of POPC (C) and PSM (D) structures. Representative snapshots showing the binding of EC, ECG dimer, and EGCG dimer on the Ld membrane (E), and surface and mass density profiles of polyphenols along the POPC lipid bilayer normal in the Ld membrane (F). For the Ld membrane, the line model (cyan) was used for POPC (yellow spheres for surface phosphorus atoms) and the vdw model (cyan) for polyphenols. The binding of EC, ECG dimer, and EGCG dimer on the Lo membrane (G), and surface and mass density profiles of polyphenols along the PSM lipid bilayer normal in the Lo membrane (H) are also shown. For the Lo membrane, the line model (blue for PSM, red for CHOL) was used for raft lipids (yellow spheres for surface phosphorus atoms, green spheres for oxygen atoms of cholesterol), and the vdw model (cyan) for polyphenols. Water molecules were removed for clarity. The snapshots were prepared by VMD 1.9.2. For the mass density profile plots, the five membrane regions (according to the scheme discussed in the text) are color-coded. Results are expressed as mean ± standard deviation (*n* = 3 independent replicates).

To further reveal where the polyphenols locate within the membrane, different regions of the lipid bilayer (Figure 3C,D) were defined by ranges of depth within the membrane, based on a scheme discussed in a previous report;^48^ the mass density profile of polyphenols along the membrane normal was calculated for the polyphenols, and these results were interpreted using this scheme. Figure 3E,F show the binding snapshots and mass density profiles of polyphenols along the bilayer normal for the Ld membrane. In the Ld membrane, the results for the mass density profiles of the different polyphenols differed significantly. The density profile of the EC molecule showed a clear peak in region 3; this indicated that EC was mainly located at the position of the lipid headgroups, in the vicinity of the glycerol ester skeleton, while the results for both the ECG and EGCG dimers showed a peak deeper within the membrane, closer to region 4, the lipid tail region. Notably, the EGCG dimer exhibited two peaks within the Ld membrane. This result probably originates from the fact that the EGCG dimer could insert itself into the POPC membrane in a V-shaped manner, as revealed by the binding snapshots (Figure 3E). The mass density profile of the EC molecule in the Lo membrane also showed a clear peak in region 3 (see Figure 3G,H), surprisingly similar to the result for the Ld membrane; however, the ECG and EGCG dimers showed completely different behavior, locating higher in the membrane than EC with the molecules positioned in regions 2 and 3. Thus, while, for the case of the Ld membrane, the ECG and EGCG dimers penetrated deeper into the loose structure of the membrane core than EC, for the case of the Lo membrane, they sat higher in the membrane, though for all three cases they can generally be seen to be sitting at roughly the position of the membrane headgroups. Given that (1) the lipids in the Ld membrane have greater flexibility due to the absence of CHOL, while the presence of CHOL in the Lo membrane increased the rigidity of the membrane^49^ and (2) the dimerized polyphenols are significantly more hydrophobic than EC (ECG dimer: logP = 5.24, EGCG dimer: logP = 4.17, EC: logP = 0.53), this result has a clear explanation: the large structure of the dimerized polyphenols results in a greater free energy barrier to penetration into the rigid Lo membrane; however, the looser structure of the Ld membrane, combined with the increased hydrophobicity of the dimerized polyphenols, results in them entering into the hydrophobic membrane core. In addition, we also explored the effects of polyphenols at different concentrations on membranes (Figure S4, Supporting Information). The results showed that dimeric polyphenols at high concentrations could self-assemble to form large aggregates, which could further exacerbate the disruptive effect on the membrane structure. The detailed results are discussed and can be viewed in the Supporting Information.

Subsequently, the time development of the minimum distance between polyphenols and membrane lipids was analyzed to reveal the binding kinetics of polyphenols in different membranes (Figures S5 and S6, Supporting Information). The minimum distance vs time (left panel) and the number of contacts between polyphenols and lipid atoms within 0.5 nm vs time (right panel) were used to quantify the lipid-binding time of polyphenols in the Ld and Lo membranes. Here, a contact is defined as any pair of atoms (one from the polyphenol and one from a lipid) separated by less than 0.16 nm.^50^ The strong lipid binding time is then defined as the cumulative time during which the number of such contacts exceeds 800. Table 2 summarizes the strong lipid binding time of polyphenols in different membranes. In the Ld membrane, the ECG dimer and EGCG dimer exhibit strong binding times of approximately 2.0 to 1.9 times as long as the case for EC. In the Lo membrane, the difference between the binding times of EC and the dimerized polyphenols is even more pronounced; the ECG dimer and EGCG dimer display binding times with PSM that are 14.4 to 10.0 times longer in comparison to EC. However, we found that there was no significant difference in the duration of strong contact between different polyphenols and CHOL. These results indicate stronger, or more efficient, binding of dimeric polyphenols (ECG dimer and EGCG dimer) than the case for EC both in the Lo and Ld membranes, with the difference in binding being more significant for the Lo membrane.

**Table 2:** Summary of Strong Lipid Binding Times (ns) of Polyphenols in Different Membranes^a^ ^,^ ^b^

membrane		polyphenols		
composition		EC	ECG dimer	EGCG dimer
Ld	POPC	109.2 ± 17.2	236.0 ± 9.1*	208.2 ± 41.1#
Lo	PSM	12.8 ± 10.4	184.7 ± 23.3*	129.9 ± 44.3*
	CHOL	1.6 ± 0.4	1.5 ± 1.0	10.6 ± 12.8

a Note: results were expressed as mean ± standard deviation (*n* = 3 independent replicates).

b # represents *p* < 0.05 (vs EC group). * represents *p* < 0.01 (vs EC group).

In addition, the results obtained in this study by separately investigating the effects of polyphenols on individual lipid membranes are highly consistent with those from our previously established system containing coexisting liquid-ordered and liquid-disordered domains. In previous work, a representative ternary model with the composition CHOL: PSM: POPC = 2:1:1 was constructed to study the interaction between polyphenols and lipid rafts,^18^ which is consistent in composition and ratio with the individual systems constructed in the present study. The results showed that dimeric polyphenols significantly disrupted the binding and hydrogen-bonding networks between CHOL and PSM, thereby disturbing the lipid raft structure. This effect strongly correlated with their inhibition of 3T3-L1 preadipocyte differentiation and underscored their role in suppressing lipid synthesis signaling pathways. However, the precise details of their actions remained unclear due to limitations of the hybrid model, which has become the focus of this study by separating the coexisting domains and examining the effects of polyphenols on each domain individually. Therefore, by combining previous findings from the coexisting membrane system with the current results from individual lipid membrane systems, both demonstrate that dimeric polyphenols exert a stronger effect on the Lo membrane than monomeric polyphenols.

### 3.4 Validation of MD Simulation Results through Langmuir Balance Measurements

To validate the results from the MD simulation, lipid monolayer experiments with a Langmuir film balance were performed. This technique could possibly show whether the tested polyphenols are miscible and have a binding preference for the membrane domains or not by measuring the changes in surface pressure–area (π–A) isotherms of lipid monolayers containing the corresponding lipids for the Ld and Lo membranes mixed with polyphenols. The π–A isotherms of polyphenols mixed into the monolayers in the Ld and the Lo states are shown in Figure 4A,B. The pure monolayer in the Ld state collapsed at a surface pressure (π) of 40.6 mN/m and a limiting mean molecular area of 84.8 Å^2^/molecule, determined by extrapolating the linear part of the isotherm in the condensed phase to zero pressure, while the pure monolayer in the Lo state collapsed at a π of 53.0 mN/m and a limiting mean molecular area of 43.1 Å^2^/molecule.

**Figure 4:**
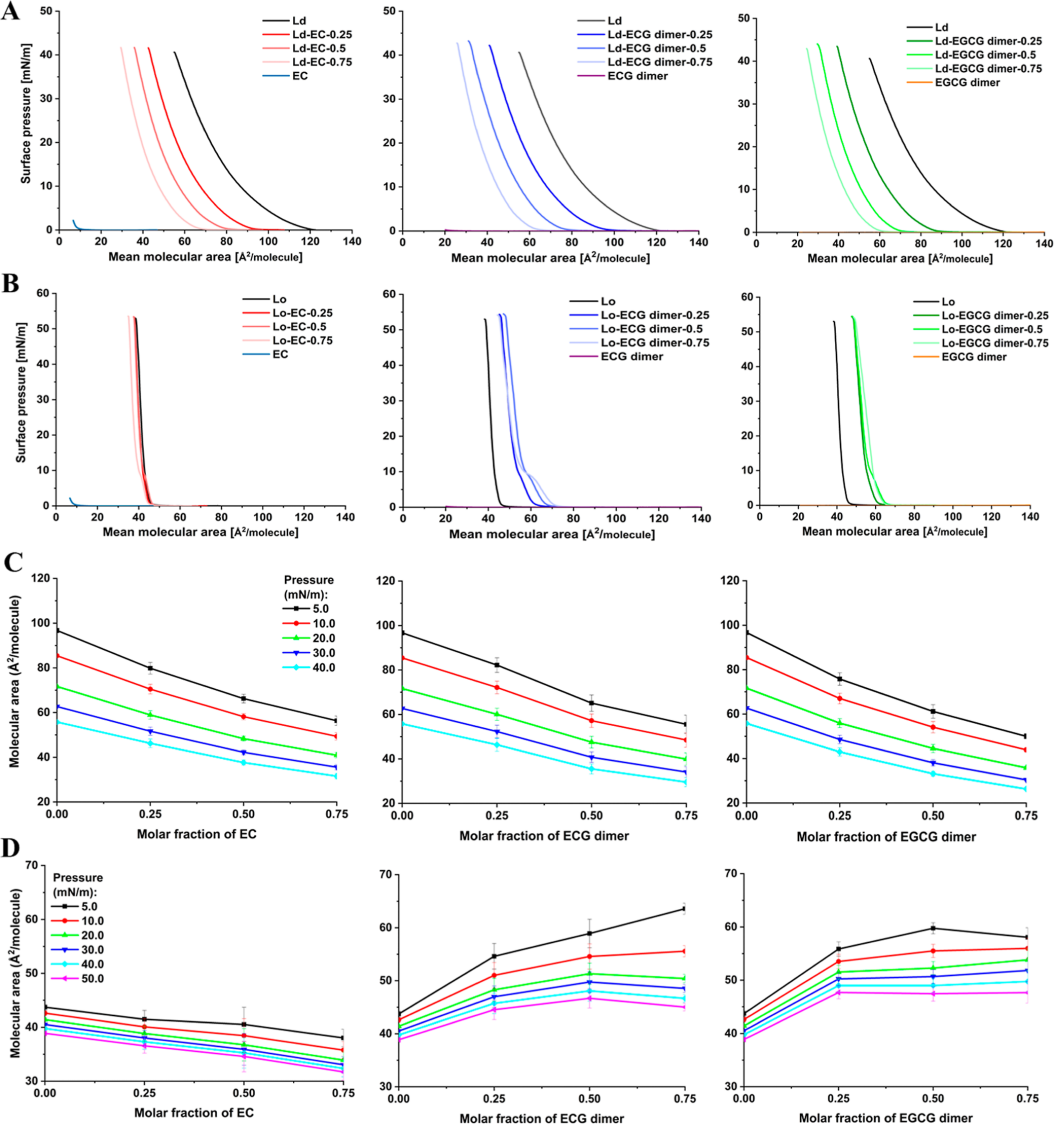
Validation of polyphenols binding to monolayers in the Ld and the Lo states through Langmuir film balance measurements. Surface pressure–area isotherms of polyphenols with monolayers in the Ld (A) or the Lo (B) states mixed with different molar fractions of polyphenols. Mean molecular area of the mixed polyphenols and the monolayers in the Ld (C) or the Lo (D) states as a function of the molar fraction of polyphenols at different surface pressures. The overall slope of the mean molecular area versus molar fraction of polyphenols at different surface pressures were used as an indicator for condensation or expansion tendency of the monolayers induced by the polyphenols (Table S4, Supporting Information). Results were expressed as mean ± standard deviation (*n* = 3 independent replicates).

Comparison shows that the π-A isotherm of the POPC monolayer (i.e., monolayer in Ld state) obtained in our study is in excellent agreement with those reported in the literature,^51,52^ confirming the reliability of our methodology. The molecular area of the POPC monolayer at the onset of collapse is typically larger than the APL in a POPC bilayer (Table 1) due to the highly compact arrangement of molecules in the monolayer. Furthermore, the literature values for POPC with an APL of 63.4 Å^2^ and a thickness of 3.92 nm^52^ closely match our simulated results of 65.1 Å^2^ and 3.90 nm, respectively (Table 1). Furthermore, the π-A isotherms show that the monolayer in the Ld state formed a loosely packed monolayer that remained in a liquid state during compression, while the monolayer in the Lo state formed a more tightly packed monolayer that reached a solid state upon compression. Thus, the monolayer in the Lo state is more rigid due to the large amount of CHOL and PSM in the monolayer, causing the higher collapse π and smaller mean molecular area of the monolayer in the Lo state compared to that of the monolayer in the Ld state.

In turn, the morphology of the monolayers in the Ld and Lo states was also imaged by Brewster angle microscopy (BAM), and the representative BAM images at low surface pressures are shown in Figure 5. More BAM images at different surface pressures are shown in the upper rows of the panels in Figures S7 and S8 (Supporting Information). From the BAM images, no phase transitions in the form of appearing domains could be observed for the pure monolayer in the Ld state, while many small and some larger domains were present in the pure monolayer in the Lo state, and these domains gradually fused into a large homogeneous monolayer along with increasing the π (from 0 to 10 mN/m).

**Figure 5:**
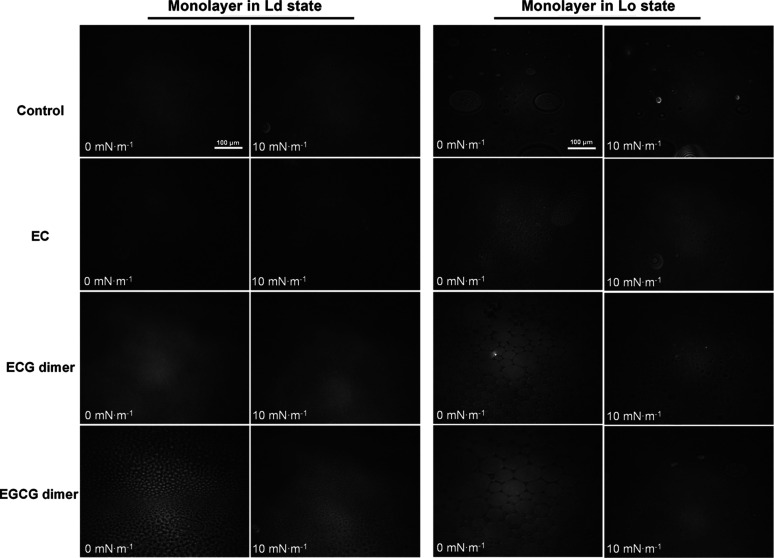
Representative BAM images of pure monolayers in the Ld/Lo state (control)­and monolayers in the Ld/Lo state mixed with polyphenols at low surface pressure values. The scale bar is 100 μm.

Subsequently, different changes in the π - A isotherms and morphology were found after the introduction of polyphenols in the different membrane monolayers. In the monolayer in the Ld state, the mean molecular area decreased upon the introduction of 0.25, 0.50, and 0.75 mol/lipid of polyphenols (EC, ECG dimer and EGCG dimer), showing a monolayer condensation effect (Figure 4A and C). Furthermore, the collapse π for the monolayer in the Ld state increased slightly with the increase in the polyphenol concentration. Also, the composition-dependent collapse π of the polyphenol mixed films provides evidence of their miscibility in the monolayer in the Ld state. The slightly higher collapse π at the corresponding concentration for dimeric polyphenols compared to that of the EC mixed film reflects that the dimeric polyphenols induce more condensed monolayers than EC, which is also seen in the larger decrease in the mean molecular area when the concentrations of the polyphenols increase in the monolayer in the Ld state (Figure 4A and C; Table S4, Supporting Information). This result was also verified by observing the formation of a larger number of small and dense bright domains after ECG dimer and EGCG dimer treatments, revealed from BAM images (Figures 5 and S7, Supporting Information).

However, an inverted tendency was observed for the monolayer in the Lo state (Figure 4B and D; Table S4, Supporting Information). The parameters of the collapse π and mean molecular area in the mixed monolayer were increased upon the introduction of 0.25, 0.50, and 0.75 mol/lipid ECG dimer and EGCG dimer, showing a monolayer expansion effect, while the mean molecular area of the mixed monolayers decreased after the introduction of EC, especially at the concentration of 0.75 mol/lipid, demonstrating a monolayer condensation effect in this case. Furthermore, the collapse π of the dimeric polyphenols in the mixed monolayers at the corresponding concentration was still higher in comparison to that of the EC mixed monolayer, indicating an increase of the extent of binding with the monolayer in the Lo state of dimeric polyphenols. From the BAM images, it could be seen that the monolayer in the Lo state in the presence of the ECG dimer and EGCG dimer formed very large and bright domains at a π of 0 mN/m (Figure 5), and these domains gradually began to fuse to form a homogeneous structure during the compression process. In contrast, the monolayer in the Lo state in the presence of EC formed a greater number of small and dense domains, as shown in Figures 5 and S8 (Supporting Information). Moreover, the phase transition observed below 10 mN/m in the monolayers in the Lo state with dimeric polyphenols (Figure 4B) indicated their segregation and self-aggregation at the interface, likely due to a molecular size mismatch with the tightly packed lipids in the monolayer in the Lo state. Rather than desorbing into the subphase, these dimeric polyphenols remain interfacially stabilized and subsequently enhance the mechanical stability of the monolayer in the Lo state against collapse at higher surface pressures by forming aggregates (Figure 4B and D). This is also consistent with the strong binding affinity (*K*_d_ = 0.3 μM for ECG dimer with Lo domain) we measured before,^13^ confirming their effective interfacial stabilization. Calculations of the compressibility modulus also confirmed that the dimeric polyphenols could fluidize the rigid monolayers in the Lo state at low surface pressures (data not shown).

Based on the presented Langmuir data, it is also important to acknowledge the inherent limitations of π-A isotherm analysis. While our results effectively demonstrate the miscibility and contrasting condensing/expanding effects of polyphenols in monolayers in Ld and Lo states, the technique primarily offers indirect thermodynamic evidence of interaction. As highlighted in the other literature,^53,54^ π-A isotherms alone cannot provide direct quantification of binding affinity or kinetic parameters. Therefore, combining other technologies will provide a more comprehensive insight into the interaction between polyphenols and cell membranes. For example, previous studies estimated the binding constant between monomeric phenols (EC) and the ordered domain of cell membrane lipids using microscale thermophoresis.^13^ The obtained binding constant was 380.6 μM, while the binding constant between dimeric polyphenols (ECG dimer) and the ordered domain of cell membrane lipids was 0.3 μM. This also indicated that dimeric polyphenols have a stronger binding ability than monomeric phenols, which is also consistent with the observed shifts in collapse pressure and molecular area in this study. To fully elucidate the affinity and kinetics suggested by our simulations and biological outcomes, future work should employ constant surface pressure penetration assays to determine definitive binding constants and penetration capacities for different membrane compositions, including monolayers in the Ld and Lo states or bilayers.

Finally, the Langmuir film balance results corroborate the MD simulations described above. First, the miscibility of polyphenols with monolayers in Ld and Lo states and the changes in collapse pressure and area indirectly reflect the differences in their binding, which mirrors very well the results obtained from the MD simulations for the corresponding membrane models. Second, MD simulations revealed that the polyphenols penetrate deeper into the looser structure of the Ld membrane than the tightly packed Lo membrane, and the large structure of the dimerized polyphenols resulted in a greater free energy barrier to penetrate into the rigid Lo membrane. This is experimentally seen as a strong condensation effect of the monolayer in the Ld state, while a slight expansion effect was seen in the case of the monolayer in the Lo state when dimeric polyphenols were present. The discrepancy between the APL values obtained from MD simulations, where the APL slightly decreased for the Lo membrane after the addition of polyphenols, and the experimental results, where the mean molecular area increased for the monolayer in the Lo state in the presence of polyphenols, originates from different study setups. In the experiments, the polyphenols were mixed with lipids and essentially incorporated into the monolayers from the start, whereas in the simulations, the polyphenols penetrated bilayers from the water phase. Nevertheless, the MD simulations indicated that dimeric polyphenols (ECG dimer and EGCG dimer) had stronger or more efficient binding to both Ld and Lo membranes than EC, with the difference in binding being more significant for the Lo membrane. This was manifested in the Langmuir film experiments as a stronger condensation and expansion tendency of the monolayers in the Ld and Lo states in the presence of dimeric polyphenols.

## 4 Conclusions

To elucidate the effect of polyphenols on lipid raft formation, in the context of the role raft domains play in 3T3-L1 preadipocyte differentiation, two different membrane models were established in this study: the liquid-ordered (Lo) membrane containing cholesterol and sphingomyelin and liquid-disordered (Ld) membrane containing phosphatidylcholine, respectively. The surface behavior and consequent interaction of dimeric polyphenols in these two different membrane models were first investigated by MD simulations. The results of a range of analysis techniques, including solvent accessible surface areas, binding snapshots, binding positions, and mass density profiles, showed that dimeric polyphenols bound to both the Ld and Lo membranes, for the case of the Ld membrane penetrating deeper within the membrane but for the case of the Lo membrane locating predominantly to the lipid headgroups. Nonetheless, we believe that the distinct binding kinetics of the polyphenols across different membranes serve as the key factor in their effect; the dimeric polyphenols exhibited significantly prolonged binding times in both Ld and Lo membranes with a more pronounced effect observed in the sphingomyelin-containing Lo membrane. This observation correlates with the disruption in lateral diffusion coefficients, as the dimeric polyphenols’ binding to the membrane was found to either constrain or induce the lipid lateral diffusion in the membrane, as measured by a change in the lateral diffusion coefficients. Finally, lipid monolayer experimental studies using the Langmuir film balance technique were used to validate the results of the simulations. An increase in the binding with both monolayers in the Ld and Lo states of dimeric polyphenols was seen, with the interaction between dimeric polyphenols and the monolayers in the Ld or Lo states found to be stronger than that of EC. In summary, we postulate that the dimeric polyphenols locate preferentially to the Lo membrane to either reduce lipid mobility or disrupt lipid packing in a way that increases lipid mobility. Through these effects on membrane behavior, the dimeric polyphenols may interfere with the signaling function and inhibit 3T3-L1 preadipocyte differentiation.

## Supplementary Material



## Data Availability

Data will be made available upon request.

## Notes

Lateral diffusion of the lipids, distributions of average lipid lateral diffusion coefficients on the same membrane leaflet as polyphenols, relationship between lateral diffusion coefficient and number of contacts, overview of interactions between high concentration of polyphenols and membranes, minimum distance analysis, BAM images, simulation system composition of polyphenol mixed Ld domain and Lo domain, time-averaged numbers of hydrogen bonds, and slope of mean molecular area vs molar fraction of polyphenols (PDF)

## References

[1] Zhang Y., Balasooriya H., Sirisena S., Ng K. (2023). The effectiveness of
dietary polyphenols in obesity management: A systematic review and
meta-analysis of human clinical trials. Food
Chem..

[2] Jin-Feng F., Ping-Ping W., Xiong F., Chin-Ping T., Chun C., Yun-Ping P. (2025). Dietary Polyphenols
in Obesity Management:
Critical Insights on Health Benefits and Mechanisms of Action. J. Food Sci..

[3] Zhu W., Zou B., Nie R., Zhang Y., Li C.-m. (2015). A-type ECG and EGCG
dimers disturb the structure of 3T3-L1 cell membrane and strongly
inhibit its differentiation by targeting peroxisome proliferator-activated
receptor γ with miR-27 involved mechanism. J. Nutr. Biochem..

[4] Wang R., Zhu W., Dang M., Deng X., Shi X., Zhang Y., Li K., Li C. (2022). Targeting Lipid Rafts
as a Rapid Screening Strategy
for Potential Antiadipogenic Polyphenols along with the Structure–Activity
Relationship and Mechanism Elucidation. J. Agric.
Food Chem..

[5] Zhu W., Deng X., Peng J., Zou B., Li C. (2017). A-type ECG
and EGCG dimers inhibit 3T3-L1 differentiation by binding to cholesterol
in lipid rafts. J. Nutr. Biochem..

[6] Mamun M. A. A., Rakib A., Mandal M., Kumar S., Singla B., Singh U. P. (2024). Polyphenols: Role in Modulating Immune
Function and
Obesity. Biomolecules.

[7] Fan J. Y., Carpentier J.-L., Van Obberghen E., Grunfeld C., Gorden P., Orci L. (1983). Morphological
changes of the 3T3-L1 fibroblast plasma membrane upon
differentiation to the adipocyte form. J. Cell
Sci..

[8] Warda M., Tekin S., Gamal M., Khafaga N., Çelebi F., Tarantino G. (2025). Lipid rafts:
novel therapeutic targets for metabolic,
neurodegenerative, oncological, and cardiovascular diseases. Lipids Health Dis..

[9] Simons K., Toomre D. (2000). Lipid rafts and signal
transduction. Nat. Rev. Mol. Cell Biol..

[10] Huo H., Guo X., Hong S., Jiang M., Liu X., Liao K. (2003). Lipid rafts/caveolae
are essential for insulin-like growth factor-1 receptor signaling
during 3T3-L1 preadipocyte differentiation induction. J. Biol. Chem..

[11] Hong S., Huo H., Xu J., Liao K. (2004). Insulin-like growth factor-1 receptor
signaling in 3T3-L1 adipocyte differentiation requires lipid rafts
but not caveolae. Cell Death Differ..

[12] Sánchez-Wandelmer J., Davalos A., Herrera E., Giera M., Cano S., de la Pena G., Lasuncion M. A., Busto R. (2009). Inhibition of cholesterol
biosynthesis disrupts lipid raft/caveolae and affects insulin receptor
activation in 3T3-L1 preadipocytes. Biochim
Biophys Acta.

[13] Wang R., Shi X., Li C. (2023). Insights into the Surface
Binding and Structural Interference
of Polyphenols with the Membrane Raft Domains in Relation to Their
Distinctive Ability to Inhibit Preadipocyte Differentiation in 3T3-L1
Cells. J. Agric. Food Chem..

[14] Wang R., Peng J., Shi X., Cao S., Xu Y., Xiao G., Li C. (2022). Change in membrane fluidity induced
by polyphenols is highly dependent on the position and number of galloyl
groups. Biochim Biophys Acta.

[15] Dutta A., Kumari M., Kashyap H. K. (2025). Tracking Cholesterol
Flip-Flop in
Mammalian Plasma Membrane through Coarse-Grained Molecular Dynamics
Simulations. Langmuir.

[16] Zhang Y., Lou J. (2025). Impact of lipid asymmetry
on membrane biophysical properties: Insights
from molecular dynamics simulations. Quant.
Biol..

[17] Niemelä P. S., Ollila S., Hyvönen M. T., Karttunen M., Vattulainen I. (2007). Assessing the nature of lipid raft
membranes. PLoS Comput. Biol..

[18] Wang R., Dang M., Zhu W., Li C. (2021). Galloyl Group in B-type
proanthocyanidin dimers was responsible for its differential inhibitory
activity on 3T3-L1 preadipocytes due to the strong lipid raft-perturbing
potency. J. Agric. Food Chem..

[19] Lee J., Cheng X., Swails J. M., Yeom M. S., Eastman P. K., Lemkul J. A., Wei S., Buckner J., Jeong J. C., Qi Y. (2016). CHARMM-GUI
Input Generator for NAMD, GROMACS, AMBER,
OpenMM, and CHARMM/OpenMM Simulations Using the CHARMM36 Additive
Force Field. J. Chem. Theory Comput..

[20] Klauda J. B., Venable R. M., Freites J. A., O’Connor J. W., Tobias D. J., Mondragon-Ramirez C., Vorobyov I., MacKerell A. D., Pastor R. W. (2010). Update
of the CHARMM All-Atom Additive
Force Field for Lipids: Validation on Six Lipid Types. J. Phys. Chem. B.

[21] Lim J. B., Rogaski B., Klauda J. B. (2012). Update of the Cholesterol Force Field
Parameters in CHARMM. J. Phys. Chem. B.

[22] Venable R. M., Sodt A. J., Rogaski B., Rui H., Hatcher E., MacKerell A. D., Pastor R. W., Klauda J. B. (2014). CHARMM
All-Atom
Additive Force Field for Sphingomyelin: Elucidation of Hydrogen Bonding
and of Positive Curvature. Biophys. J..

[23] Jorgensen W. L., Chandrasekhar J., Madura J. D., Impey R. W., Klein M. L. (1983). Comparison
of simple potential functions for simulating liquid water. J. Chem. Phys..

[24] MacKerell A. D., Bashford D., Bellott M., Dunbrack R. L., Evanseck J. D., Field M. J., Fischer S., Gao J., Guo H., Ha S. (1998). All-atom empirical potential for molecular modeling
and dynamics studies of proteins. J. Phys. Chem.
B.

[25] Abraham M. J., Murtola T., Schulz R., Páll S., Smith J. C., Hess B., Lindahl E. (2015). GROMACS: High
performance
molecular simulations through multi-level parallelism from laptops
to supercomputers. SoftwareX.

[26] Buchoux S. (2017). FATSLiM: a
fast and robust software to analyze MD simulations of membranes. Bioinformatics.

[27] Smith P., Lorenz C. D. (2021). LiPyphilic: A Python Toolkit for
the Analysis of Lipid
Membrane Simulations. J. Chem. Theory Comput..

[28] Michaud-Agrawal N., Denning E. J., Woolf T. B., Beckstein O. (2011). MDAnalysis:
A toolkit for the analysis of molecular dynamics simulations. J. Comput. Chem..

[29] Gowers, R. ; Linke, M. ; Barnoud, J. ; Reddy, T. ; Melo, M. ; Seyler, S. ; Dotson, D. ; Domanski, J. ; Buchoux, S. ; Kenney, I. ; Beckstein, O. MDAnalysis: a Python package for the rapid analysis of molecular dynamics simulations; Proceedings of the Python in Science Conference; SciPy, 2016.pp 98–105.

[30] Frisch, M. J. ; Gaussian 09 Reference; Revision B. 01, Gaussian, Inc, 2009, Wallingford, USA.

[31] Wang J., Cieplak P., Kollman P. A. (2000). How well
does a restrained electrostatic
potential (RESP) model perform in calculating conformational energies
of organic and biological molecules?. J. Comput.
Chem..

[32] Vanommeslaeghe K., Hatcher E., Acharya C., Kundu S., Zhong S., Shim J., Darian E., Guvench O., Lopes P., Vorobyov I. (2010). CHARMM
general force field: A force field for
drug-like molecules compatible with the CHARMM all-atom additive biological
force fields. J. Comput. Chem..

[33] Bussi G., Donadio D., Parrinello M. (2007). Canonical
sampling through velocity
rescaling. J. Chem. Phys..

[34] Parrinello M., Rahman A. (1981). Polymorphic transitions in single crystals: A new molecular
dynamics method. J. Appl. Phys..

[35] Hess B., Bekker H., Berendsen H. J. C., Fraaije J. G. E. M. (1997). LINCS: A linear
constraint solver for molecular simulations. J. Comput. Chem..

[36] Essmann U., Perera L., Berkowitz M. L., Darden T., Lee H., Pedersen L. G. (1995). A smooth particle
mesh Ewald method. J. Chem. Phys..

[37] Hockney R. W., Goel S. P., Eastwood J. W. (1974). Quiet high-resolution
computer models
of a plasma. J. Comput. Phys..

[38] Humphrey W., Dalke A., Schulten K. (1996). VMD: visual
molecular dynamics. J. Mol. Graphics.

[39] Smith P., Ziolek R. M., Gazzarrini E., Owen D. M., Lorenz C. D. (2019). On the
interaction of hyaluronic acid with synovial fluid lipid membranes. Phys. Chem. Chem. Phys..

[40] Dong X.-q., Zou B., Zhang Y., Ge Z.-z., Du J., Li C.-m. (2013). Preparation
of A-type proanthocyanidin dimers from peanut skins and persimmon
pulp and comparison of the antioxidant activity of A-type and B-type
dimers. Fitoterapia.

[41] Kučerka N., Tristram-Nagle S., Nagle J. F. (2006). Structure of Fully
Hydrated Fluid
Phase Lipid Bilayers with Monounsaturated Chains. J. Membr. Biol..

[42] Wang L., Murphy-Ullrich J. E., Song Y. (2014). Molecular insight into
the effect
of lipid bilayer environments on thrombospondin-1 and calreticulin
interactions. Biochemistry.

[43] Slotte J. P. (2016). The importance
of hydrogen bonding in sphingomyelin’s membrane interactions
with co-lipids. Biochim Biophys Acta.

[44] Saito H., Shinoda W. (2011). Cholesterol Effect
on Water Permeability through DPPC
and PSM Lipid Bilayers: A Molecular Dynamics Study. J. Phys. Chem. B.

[45] Filippov A., Orädd G., Lindblom G. (2006). Sphingomyelin structure influences
the lateral diffusion and raft formation in lipid bilayers. Biophys. J..

[46] Mei J., Liu C., Yang H., Ma X., Ai H. (2022). Molecular
mechanisms
of direct and indirect interplay between amyloid β42 oligomer
and characteristic lipids. ChemPhysMater.

[47] Lehtinen J., Magarkar A., Stepniewski M., Hakola S., Bergman M., Róg T., Yliperttula M., Urtti A., Bunker A. (2012). Analysis of
cause of failure of new targeting peptide in PEGylated liposome: Molecular
modeling as rational design tool for nanomedicine. Eur. J. Pharm. Sci..

[48] Róg T., Girych M., Bunker A. (2021). Mechanistic Understanding
from Molecular
Dynamics in Pharmaceutical Research 2: Lipid Membrane in Drug Design. Pharmaceuticals.

[49] Róg T., Vattulainen I. (2014). Cholesterol,
sphingolipids, and glycolipids: What do
we know about their role in raft-like membranes?. Chem. Phys. Lipids.

[50] Pham T., Cheng K. H. (2022). Exploring the binding
kinetics and behaviors of self-aggregated
beta-amyloid oligomers to phase-separated lipid rafts with or without
ganglioside-clusters. Biophys. Chem..

[51] Grauby-Heywang C., Moroté F., Mathelié-Guinlet M., Gammoudi I., Faye N. R., Cohen-Bouhacina T. (2016). Influence
of oxidized lipids on palmitoyl-oleoyl-phosphatidylcholine
organization, contribution of Langmuir monolayers and Langmuir–Blodgett
films. Chem. Phys. Lipids.

[52] Żak A., Korshunova K., Rajtar N., Kulig W., Kepczynski M. (2023). Deciphering
Lipid Arrangement in Phosphatidylserine/Phosphatidylcholine Mixed
Membranes: Simulations and Experiments. Langmuir.

[53] de
Matos Alves Pinto L., Malheiros S. V., Lino A. C., de Paula E., Perillo M. A. (2006). Hydroxyzine, promethazine and thioridazine interaction
with phospholipid monomolecular layers at the air-water interface. Biophys. Chem..

[54] Noe M. M., Rodríguez J. A., Barredo Vacchelli G.
R., Camperi S. A., Franchi A. N., Turina A. V., Perillo M. A., Nolan V. (2025). Whey-Derived
Antimicrobial Anionic Peptide Interaction with Model Membranes and
Cells. Langmuir.

