# Supplementary material for: Dimeric Polyphenol Effect on Liquid-Ordered and Liquid-Disordered Membranes: Combined Insights from Molecular Dynamics Simulation and Langmuir Balance Measurements
Source: Langmuir. 2026 Mar 20;42(13):9021–33. doi: 10.1021/acs.langmuir.5c05280 (PMC13063806; doi:10.1021/acs.langmuir.5c05280)
Supplement: Supplementary file 1 [file la5c05280_si_001.pdf]

Supporting information:

Dimeric polyphenol effect on liquid-ordered and liquid-disordered membranes: combined insight from molecular dynamics simulation and Langmuir balance measurements

Ruifeng Wang[a,b,c], Suvi Heinonen[b], Elina Vuorimaa-Laukkanen[d], Chunmei Li[a], Tapani Viitala[e,f], and Alex Bunker[b*]

[a] College of Food Science and Technology, Huazhong Agricultural University, Wuhan, Hubei 430070, China

[b] Division of Pharmaceutical Biosciences, Faculty of Pharmacy, University of Helsinki, Helsinki 00014, Finland

[c] State Key Laboratory of Quality Research in Chinese Medicine, Institute of Chinese Medical Sciences, University of Macau, Macau 999078, China

[d] Chemistry and Advanced Materials, Faculty of Engineering and Natural Sciences, Tampere University, Tampere 33720, Finland

[e] Division of Pharmaceutical Chemistry and Technology, Faculty of Pharmacy, University of Helsinki, Helsinki 00014, Finland

[f] Pharmaceutical Sciences Laboratory, Faculty of Science and Engineering, Åbo Akademi University, Turku 20520, Finland

* Corresponding author. Email: Alex.bunker@helsinki.fi

Supplementary results on lateral diffusion of the lipids

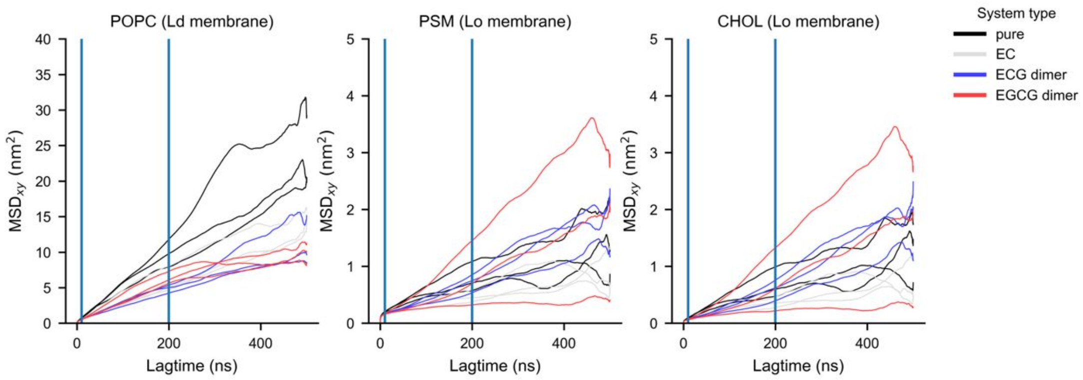

Figure S1. Mean square displacement curves for different lipids in the Ld and Lo membranes with (EC, ECG dimer, EGCG dimer) and without (pure) the presence of various polyphenols. All lipids in both membrane leaflets have been considered in the analysis. Each replicate for a similar type of system is represented with the same coloring. The start and end points for the linear fit are denoted with blue lines.

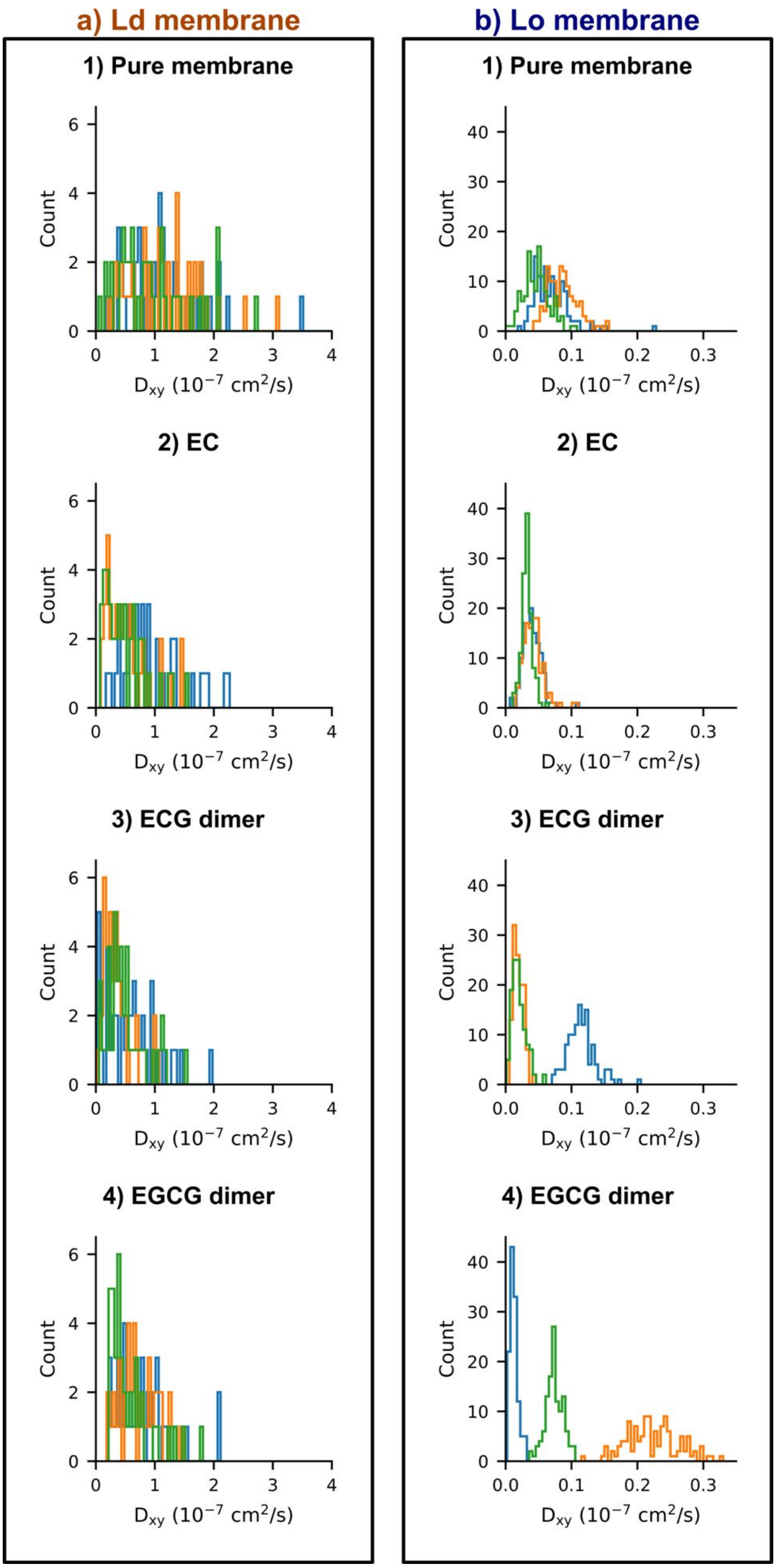

a) Ld membrane
b) Lo membrane
1) Pure membrane
2) EC
3) ECG dimer
4) EGCG dimer
Count
D_{xy} (10^{-7} cm^2/s)

Figure S2. Distribution of the average lipid lateral diffusion coefficients on the same membrane leaflet as polyphenol calculated based on the final 500 ns of simulations for (a) the Ld membrane and (b) the Lo membrane. Each replicate has been colored with distinctive colors (blue=1, orange=2, green=3) to clarify the differences between the distributions.

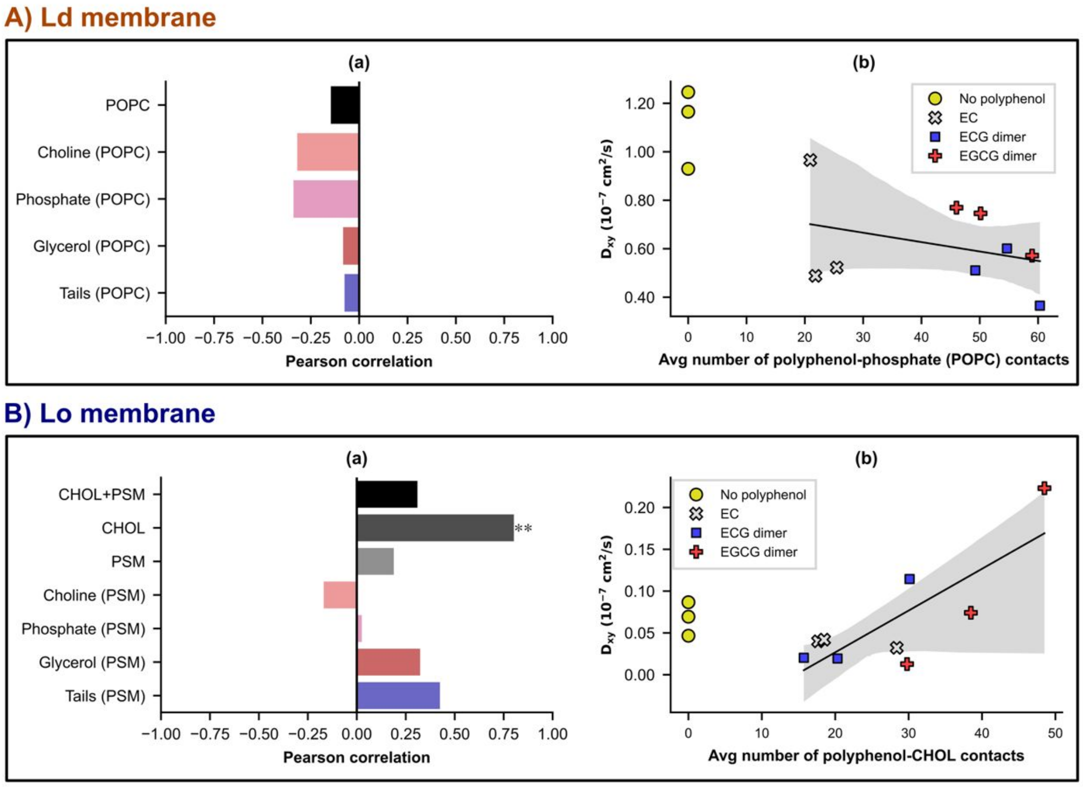

Figure S3. Relationship between the average lateral diffusion of lipids on the same leaflet as the polyphenol and the average number of polyphenol-lipid heavy-atom contacts within a 4.5 Å cutoff (*i.e.,* D_{xy} vs. $N_{contacts}$) for the A) Ld and B) Lo membranes. Only systems containing polyphenols were included in the correlation analysis (n = 9). Panel (a) shows the Pearson correlations between D_{xy} and $N_{contacts}$ for different lipids or lipid subregions. The lipid subregions listed for PSM and POPC correspond to those in Figure 3D, and two asterisks (∗∗) denote statistically significant correlations ($p < 0.01$). Pearson correlations for the Ld membrane: POPC: -0.15 ($p = 0.71$); choline: -0.32 ($p = 0.40$); phosphate: -0.34 ($p = 0.37$); glycerol: -0.08 ($p = 0.83$); tails: -0.08 ($p = 0.85$). D_{xy} vs. $N_{contacts}$ Pearson correlations for the Lo membrane: all lipids: 0.31 ($p = 0.42$); CHOL: 0.80 ($p < 0.01$); PSM: 0.19 ($p = 0.63$); choline: -0.17 ($p = 0.66$); phosphate: 0.03 ($p = 0.95$); glycerol: 0.32 ($p = 0.40$), tails: 0.42 ($p = 0.25$). Panel (b) presents the average D_{xy} plotted against the average number of contacts for the lipid or lipid subregion that produced the strongest D_{xy} vs. $N_{contacts}$ correlation in each independent simulation. Linear regression fits with 95% confidence intervals are shown for the polyphenol-containing systems, and the corresponding diffusion coefficient values from membrane-only systems are included as yellow circles for reference.

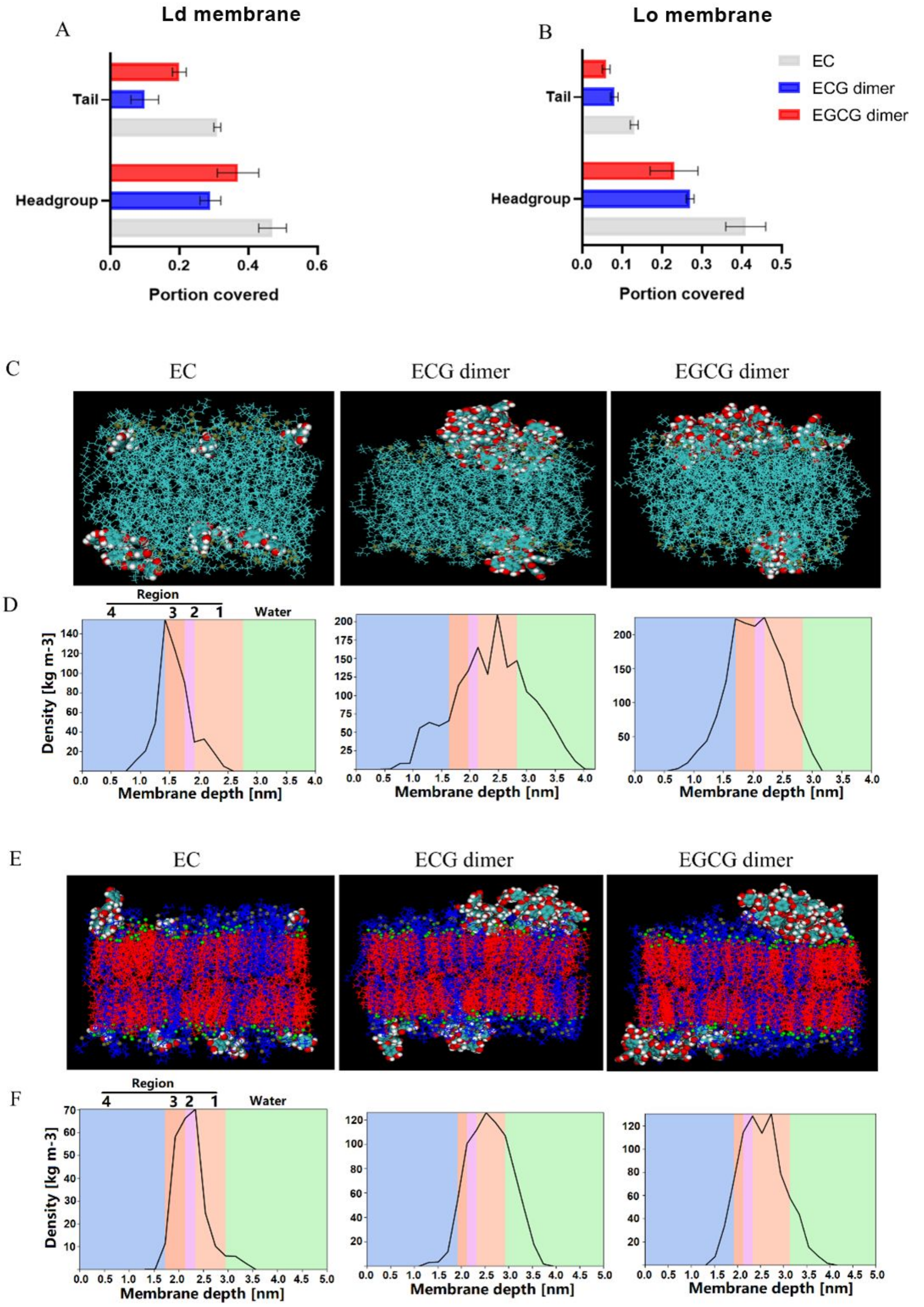

Figure S4. Overview of interactions between a high concentration of polyphenols and Ld as well as Lo membranes. Surface area of a high concentration of polyphenols covering the headgroup and tails of lipids in the Ld (A) and the Lo (B) membranes. Representative snapshots showing the binding of EC, ECG dimer, and EGCG dimer at high concentration on the Ld membrane (C) surface and mass density profiles of polyphenols along the POPC lipid bilayer in the Ld membrane (D). For the Ld membrane, the line model (cyan) was used for POPC (yellow spheres for surface phosphorus atoms) and vdw model (cyan) for polyphenols. The binding of EC, ECG dimer, and EGCG dimer at the high concentration on the Lo membrane (E), surface and mass density profiles of polyphenols along the PSM lipid bilayer in the Lo membrane (F) are also shown. For the Lo membrane, the line model (blue for PSM, red for CHOL) was used for raft lipids (yellow spheres for surface phosphorus

atoms, green spheres for oxygen atoms of cholesterol) and vdw model (cyan) for polyphenols. Water molecules were removed for clarity. The snapshots were prepared by VMD 1.9.2. Results are expressed as mean ± standard deviation (n = 3 independent replicates).

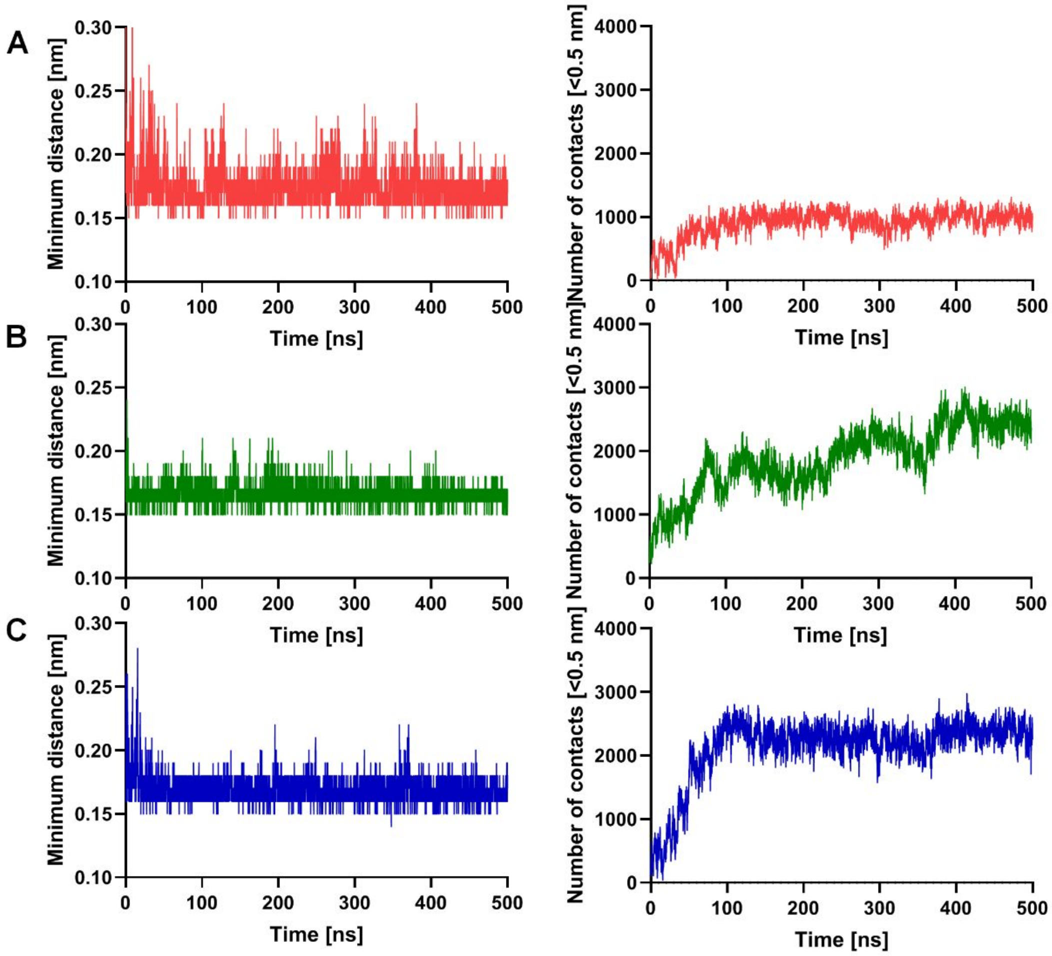

Figure S5. Minimum distance analysis of polyphenols binding to the Ld membrane. Two-panel plots of polyphenols-lipid minimum distance analysis. The left panel shows minimum distance vs. time, and the right panel shows the number of contacts between polyphenols and POPC atoms within 0.5 nm vs. time. All data are displayed in color based on the polyphenol types with EC (red), ECG dimer (green), and EGCG dimer (blue).

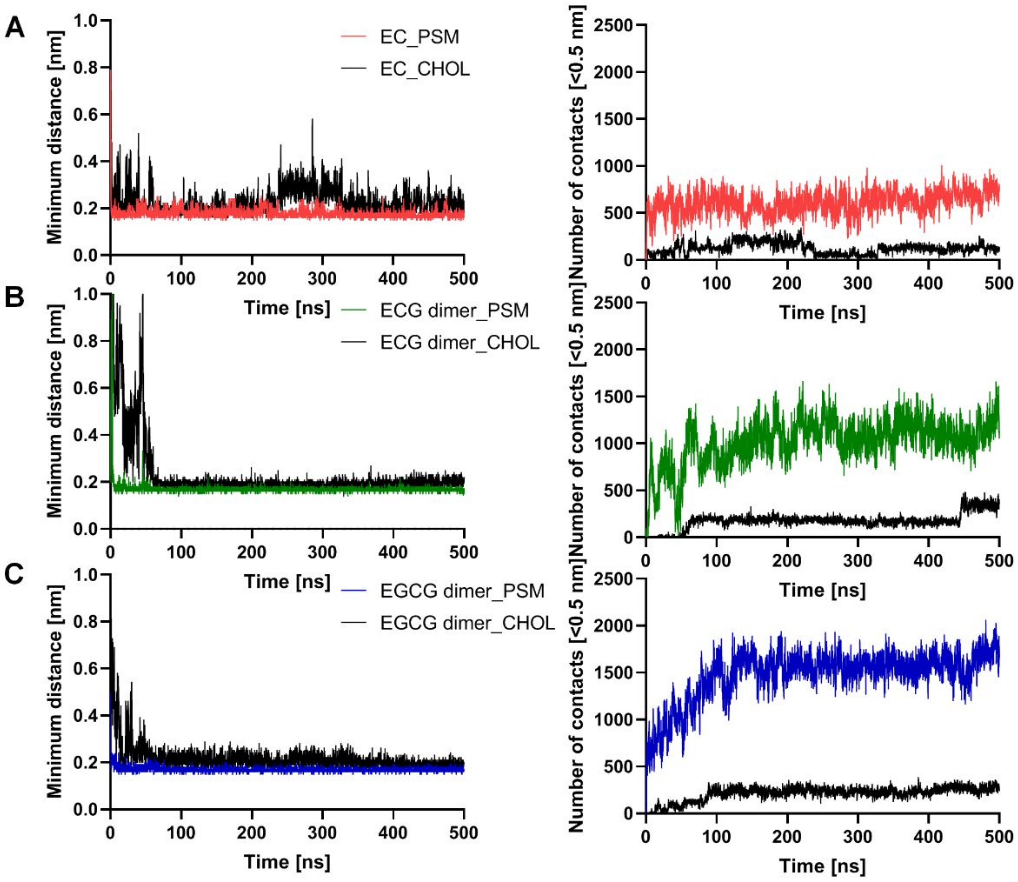

Figure S6. Minimum distance analysis of polyphenols binding to the Lo membrane. Two-panel plots of polyphenols-lipid minimum distance analysis. The left panel shows minimum distance vs. time, and the right panel shows the number of contacts between polyphenols and PSM/CHOL atoms within 0.5 nm vs. time. All data are displayed in color based on the polyphenol types with EC (red), ECG dimer (green), and EGCG dimer (blue). The data points with CHOL are shown in black.

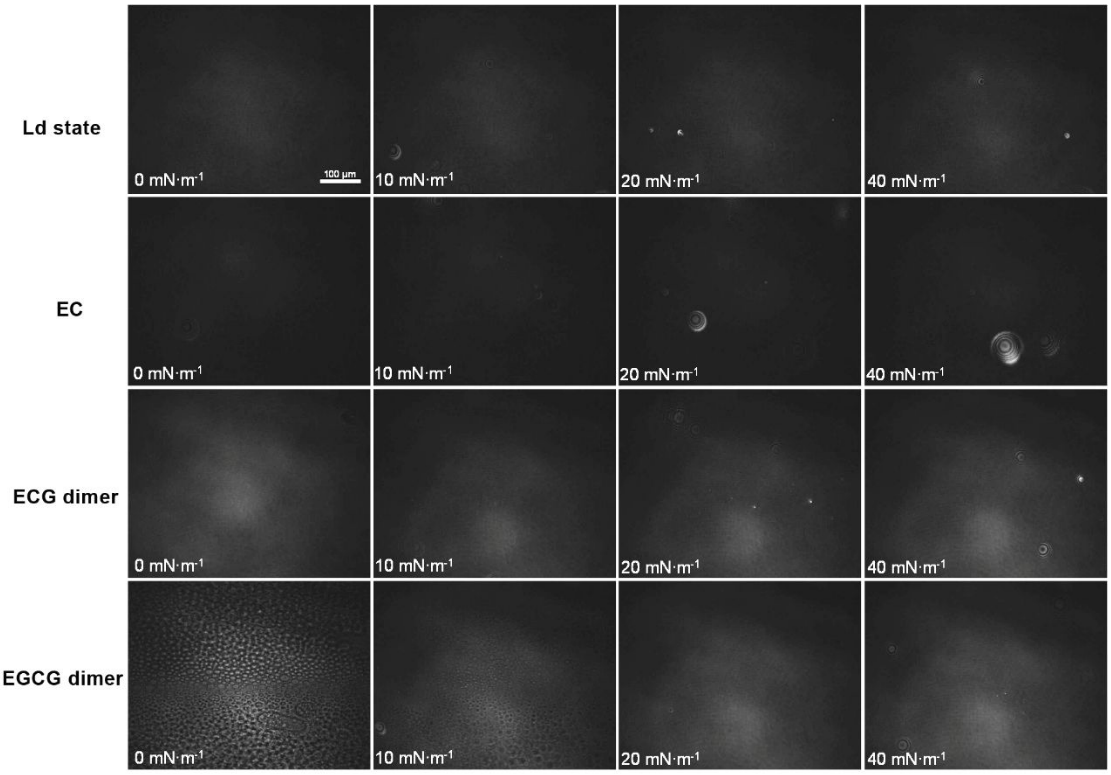

Figure S7. BAM images of monolayers in the Ld state and mixed films formed by monolayers in the Ld state and polyphenols at given surface pressure values. The scale bar is 100 µm.

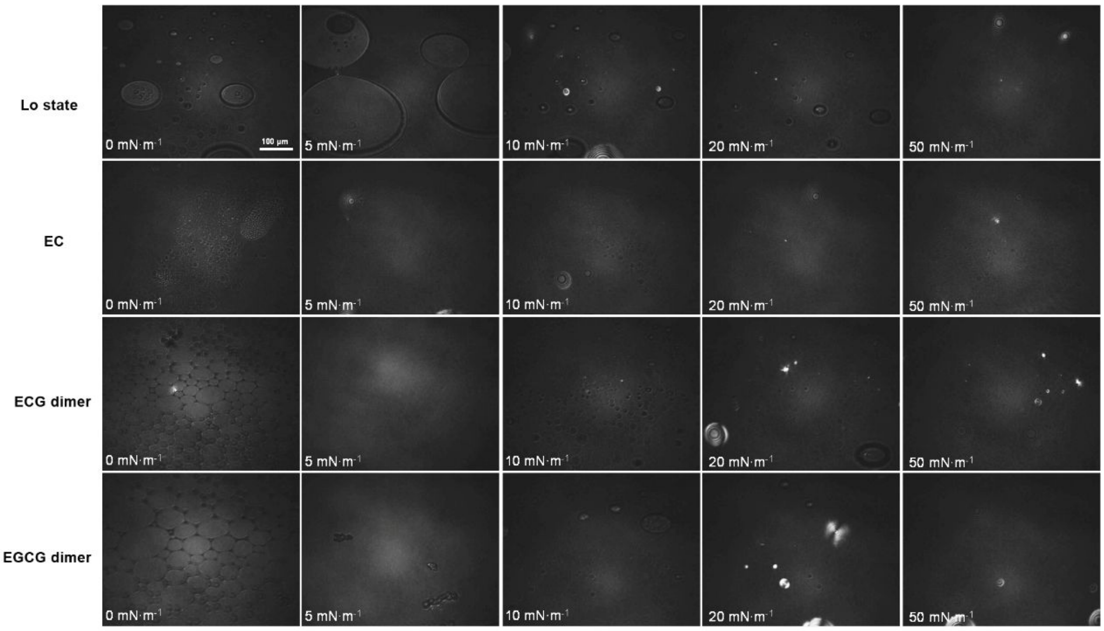

Figure S8. BAM images of monolayers in the Lo state and mixed films formed by monolayers in the Lo state and polyphenols at given surface pressure values. The scale bar is 100 µm.

Table S1. Simulation system composition of polyphenol mixed Ld membranes.

System	Ld membrane<br>Number of molecules (POPC)		EC	ECG dimer	EGCG dimer	Water	Total atom
	Upper leaflet	Lower leaflet					
Ld-EC	40	40	1	-	-	4384	23907
Ld-ECG dimer	40	40	-	1	-	4348	23860
Ld-EGCG dimer	40	40	-	-	1	4350	23868

Note: box size: 4.9 × 4.9 × 10.0 nm

Table S2. Simulation system composition of polyphenol mixed Lo membrane.

System	Lo membrane<br>Number of molecules (CHOL)		Number of molecules (PSM)		EC	ECG dimer	EGCG dimer	Wate r	Total atom
	Upper leaflet	Lower leaflet	Upper leaflet	Lower leaflet					
Lo-EC	80	80	40	40	1	-	-	9613	50874
Lo-ECG dimer	80	80	40	40	-	1	-	9579	50833
Lo-EGCG dimer	80	80	40	40	-	-	1	9579	50835

Note: box size: 6.9 × 6.9 × 11.0 nm

Table S3. Summary of the time-averaged number of hydrogen bonds within the currently studied Ld and Lo membranes. Both polyphenol-lipid and lipid-lipid hydrogen bonds are presented. Both intermolecular (= intermol.) and intramolecular (= intramol.) hydrogen bonds have been quantified separately for PSM-PSM.

	Ld membrane			Lo membrane		
Polyphenol composition	polyphenol - POPC	polyphenol - PSM	polyphenol - CHOL	PSM-PSM (intermol.)	PSM-PSM (intramol.)	PSM-CHOL
				9.31 ± 0.66	43.09 ± 0.31	30.18 ± 0.42
EC	2.06 ± 0.62	1.68 ± 0.52	0.59 ± 0.17	7.71 ± 1.70	43.66 ± 0.49	32.50 ± 0.69
ECG dimer	4.25 ± 1.16	3.00 ± 0.90	0.36 ± 0.22	7.92 ± 1.28	43.33 ± 0.43	32.19 ± 2.19
EGCG dimer	4.39 ± 1.14	2.36 ± 0.88	1.34 ± 0.25	7.90 ± 1.26	43.71 ± 0.55	32.65 ± 1.60

Note: Results were expressed as mean ± standard deviation (n = 3 independent replicates).

Table S4. The overall slope of the linear fit to the mean molecular area versus molar fraction of polyphenols at different surface pressures taken as an indicator for condensation or expansion tendency of the monolayers induced by the polyphenols. The larger the absolute value is the stronger the tendency.

**Monolayers in Ld state mixed with -polyphenols**			
Pressure (mN/m)	EC	ECG dimer	EGCG dimer
5	-56.1	-57.8	-62.4
10	-50.4	-51.8	-55.6
20	-43.0	-44.2	-48.0
30	-38.0	-40.0	-43.2
40	-33.6	-36.4	-40.0
**Monolayers in the Lo state mixed with-polyphenols**			
Pressure (mN/m)	EC	ECG dimer	EGCG dimer
5	-7.6	26.9	27.0
10	-9.1	18.0	23.3
20	-10.0	12.7	21.5
30	-9.9	11.3	20.3
40	-9.8	9.7	18.4
50	-9.4	8.8	17.1
